# Understanding the capacity of children with congenital unilateral below-elbow deficiency to actuate their affected muscles

**DOI:** 10.1038/s41598-024-54952-7

**Published:** 2024-02-24

**Authors:** Marcus A. Battraw, Justin Fitzgerald, Michelle A. James, Anita M. Bagley, Wilsaan M. Joiner, Jonathon S. Schofield

**Affiliations:** 1 Department of Mechanical and Aerospace Engineering, University of California, Davis, Davis, CA USA; 2 Department of Biomedical Engineering, University of California, Davis, Davis, CA USA; 3 Department of Neurobiology, Physiology and Behavior, University of California, Davis, Davis, CA USA; 4 Clinical and Translational Science Center, University of California, Davis Health, Sacramento, CA USA; 5 Shriners Children’s – Northern California, Sacramento, CA USA; 6 Department of Orthopaedic Surgery, University of California, Davis Health, Sacramento, CA USA; 7 Department of Neurology, University of California, Davis Health, Sacramento, CA USA

**Keywords:** Paediatric research, Translational research

## Abstract

In recent years, commercially available dexterous upper limb prostheses for children have begun to emerge. These devices derive control signals from surface electromyography (measure of affected muscle electrical activity, sEMG) to drive a variety of grasping motions. However, the ability for children with congenital upper limb deficiency to actuate their affected muscles to achieve naturalistic prosthetic control is not well understood, as compared to adults or children with acquired hand loss. To address this gap, we collected sEMG data from 9 congenital one-handed participants ages 8–20 years as they envisioned and attempted to perform 10 different movements with their missing hands. Seven sEMG electrodes were adhered circumferentially around the participant’s affected and unaffected limbs and participants mirrored the attempted missing hand motions with their intact side. To analyze the collected sEMG data, we used time and frequency domain analyses. We found that for the majority of participants, attempted hand movements produced detectable and consistent muscle activity, and the capacity to achieve this was not dissimilar across the affected and unaffected sides. These data suggest that children with congenital hand absence retain a degree of control over their affected muscles, which has important implications for translating and refining advanced prosthetic control technologies for children.

## Introduction

Approximately 1 in 500 live births will present with an upper limb deficiency^1^, which is the most common reason for limb absence in children^2^. Among those born with upper limb deficiencies, children with unilateral congenital below-elbow deficiency (UCBED) will most typically present with limb characteristics amenable to prosthesis prescription. Although there are a variety of upper limb prostheses available for these children, they are regularly abandoned with 35%–45% of prescribed devices not being used^3^. In fact, these devices often fall short of meeting the wearers’ needs and typically don’t provide sufficient function and/or improve quality of life^4^. Encouragingly, dexterous prostheses that resemble the form and function of intact hands are becoming widely available for adults and more recently for children^5,6^. These devices achieve a variety of grasping movements and as a result, hold the potential to offer additional functional benefits. However, limited research has been performed to address and refine these systems for the unique challenges and demands of children.

With increased prosthetic dexterity comes the need for more sophisticated control systems to manage the newly available function. State-of-the-art control systems for adult prostheses use machine learning to predict the user’s motor intent from patterns of muscle electrical activity (surface electromyography, sEMG) and map these predictions to corresponding prosthetic movements^7–10^. Despite promising results in adults with acquired amputation, few studies have been performed in those with congenital limb absence. One study recruited N = 4 adults with congenital limb absence and demonstrated limited success, applying sEMG and machine learning to predict 11 hand movements and finding classification accuracies of 52.1% ± 15.0%^11^. Additionally, in a cohort of children (N = 5, < 21 years old) and adults (N = 2) with UCBED, a commercially available control system was used to predict missing limb movements^12^. Such systems have been primarily designed for those with acquired limb loss and have yet to be refined for individuals with congenital limb differences. Only 2 of the 5 children demonstrated a classification accuracy greater than 80% for a limited repertoire of only 3 degrees of freedom^12^. Of these two publications, one studied and recruited children in a limited setting; however, an adult-specific prosthesis control system was employed which is unlikely to be directly applicable to children^13^. Collectively, these limitations restrict the translation of their findings to a more comprehensive understanding of affected muscle activity in children with UCBED.

The translation of advanced sEMG techniques to decode motor intent and control prostheses for children with UCBED requires a more thorough understanding of the capabilities of their affected muscles. sEMG characteristics such as root mean squared (RMS), moving average, linear envelope, mean frequency (MNF), and median frequency are often used to investigate the biological control an individual has over their limb(s)^14–17^. These characteristics have yet to be investigated in children born with limb absence and therefore the capacity for these children to actuate their affected muscles is unknown.

In this work, we investigated muscle activity using surface electromyography of children’s affected and healthy unaffected-contralateral muscles while they attempted to perform a variety of hand movements. We evaluated measures of within-limb and across-limb consistency, and distinguishability using RMS and MNF measures. Our study had three main objectives to identify if children with UCBED could consistently and distinguishably actuate their affected muscles, and to examine how this actuation might compare to their unaffected side. First, we assessed children’s ability to perform various distinguishable hand movements using split-data representational dissimilarity analysis, aiming to determine whether there was distinguishable structure in attempted movements. Second, we sought to quantify statistically significant differences in the consistency of movements by comparing the affected and unaffected limbs. Lastly, we expanded our investigation to determine if a statistically significant relationship existed in the hand movement structure between the affected and unaffected limbs using representational dissimilarity randomization analysis.

## Methods

### Participants

Nine participants (8 male, 1 female) with UCBED aged 8–20 years old participated in this study (mean = 14 years; SD = 4.4 years). Research protocols were approved by the Institutional Review Board at the Shriners Children’s—Northern California and were performed in accordance with the relevant guidelines and regulations. Participants provided written informed assent and/or their legal guardians provided written informed consent. Participant details can be found in Table 1. Additionally, participants exhibiting only a unilateral congenital upper limb deficiency in the forearm region were included. Children with UCBED otherwise underwent typical maturation and development. All potential candidates were clinically screened, and those with atypical development aside from UCBED were excluded. Therefore, we treated the data collected from the unaffected limb of each participant (described below) as a control for comparison.

**Table 1:** Demographic information for participants with unilateral congenital below-elbow deficiency.

Subject ID	Age	Sex	Affected Limb	Limb Length (cm)		Limb Circumference (cm)		Prosthesis Use
				Right	Left	Right	Left	
SHR-A	20	Male	Left		13		15	PA
SHR-B	8	Male	Right	14	16	20	21.5	PA
SHR-C	11	Male	Right	18	21	18	20	None
SHR-D	9	Male	Left	21.5	12.5	22.5	18.5	None
SHR-E	18	Male	Right	15	28	21	23	BP†
SHR-F	16	Female	Left	26.5	11.5	26.5	23.5	PA
SHR-G	19	Male	Left	26.5	13	25.5	21.5	Myo & PA
SHR-H	14	Male	Left	25	8	26	23	BP
SHR-I	12	Male	Right	10	24	21	24	BP

*PA* Passive device, *BP* body-powered device, *Myo* myoelectric device.

^†^Activity specific device.

### Data collection

A 16-Channel Delsys Trigno surface EMG System (Delsys, Natick, USA) was used to capture affected and unaffected forearm muscle activity. The system consisted of wireless Trigno Mini Sensors with an inter-electrode spacing of 10 mm and dual on-board differential references. The sEMG signal input was set to a range of ± 11 mV and the data were bandpass filtered at 20–450 Hz^18,19^. A National Instruments USB-6210 data acquisition system (National Instruments Corp., Austin, USA) sampled sEMG data at 6000 Hz and stored data in a MATLAB (MathWorks, Inc., Natick, USA) file structure for postprocessing and analysis. Additionally, a Logitech C922 high-definition camera (Logitech International S.A., Lausanne, Switzerland) sampled at 15 frames per second was synchronized with the sEMG data.

### Experiment

**Figure 1:**
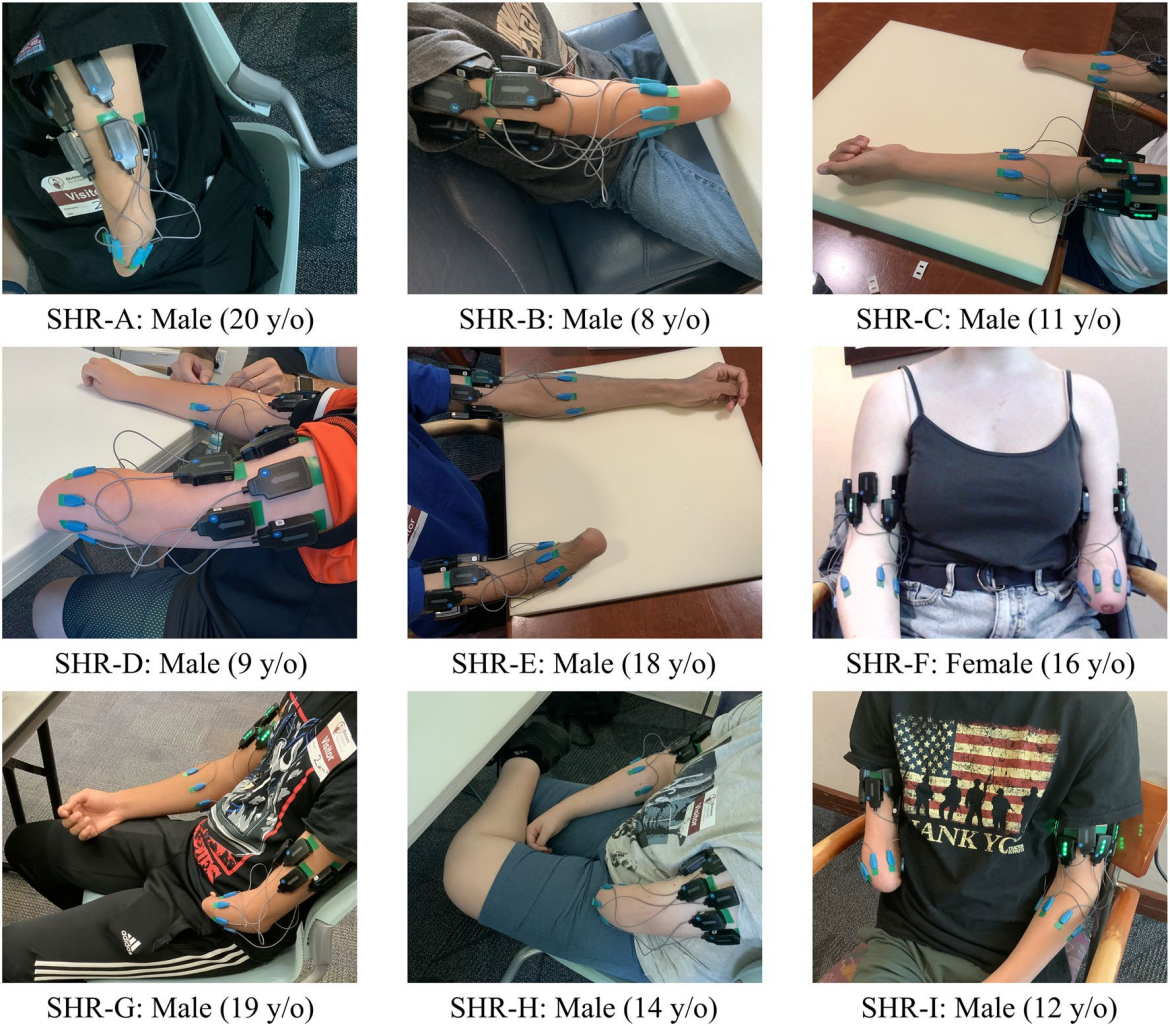
Participants with sEMG electrodes donned across unaffected and affected limbs with sex and age in years old (y/o) specified.

We first performed “conceptual training” with each participant in which we introduced our experimental equipment, described terminology used during testing, provided a simple overview of sEMG, and allowed them to familiarize themselves^20^. After, we adhered the sEMG electrodes to their skin over the affected and unaffected forearm muscles using double-sided adhesive. The participants were asked to contract their affected limb as if they were making a fist while we palpated to find where the superficial muscles on their ventral side were most firm and we placed the first sEMG electrode there. If participants had a small residual limb length, less than about 10 cm, then the first sEMG electrode was placed in the center of their residual limb starting on the ventral side. In either case, the remaining sEMG electrodes were placed around the circumference of the participants’ forearm muscles following the typical circumferential equidistant protocol^21–23^. This procedure was repeated in the unaffected limb and electrodes were placed on the most proximal two-thirds of the forearm musculature (Fig. 1). As reported in prior work, 7 sEMG electrodes were used on each side^24^ (with the exception of SHR-A’s affected limb where 4 sEMG electrodes were adhered due to size limitations).

**Figure 2:**
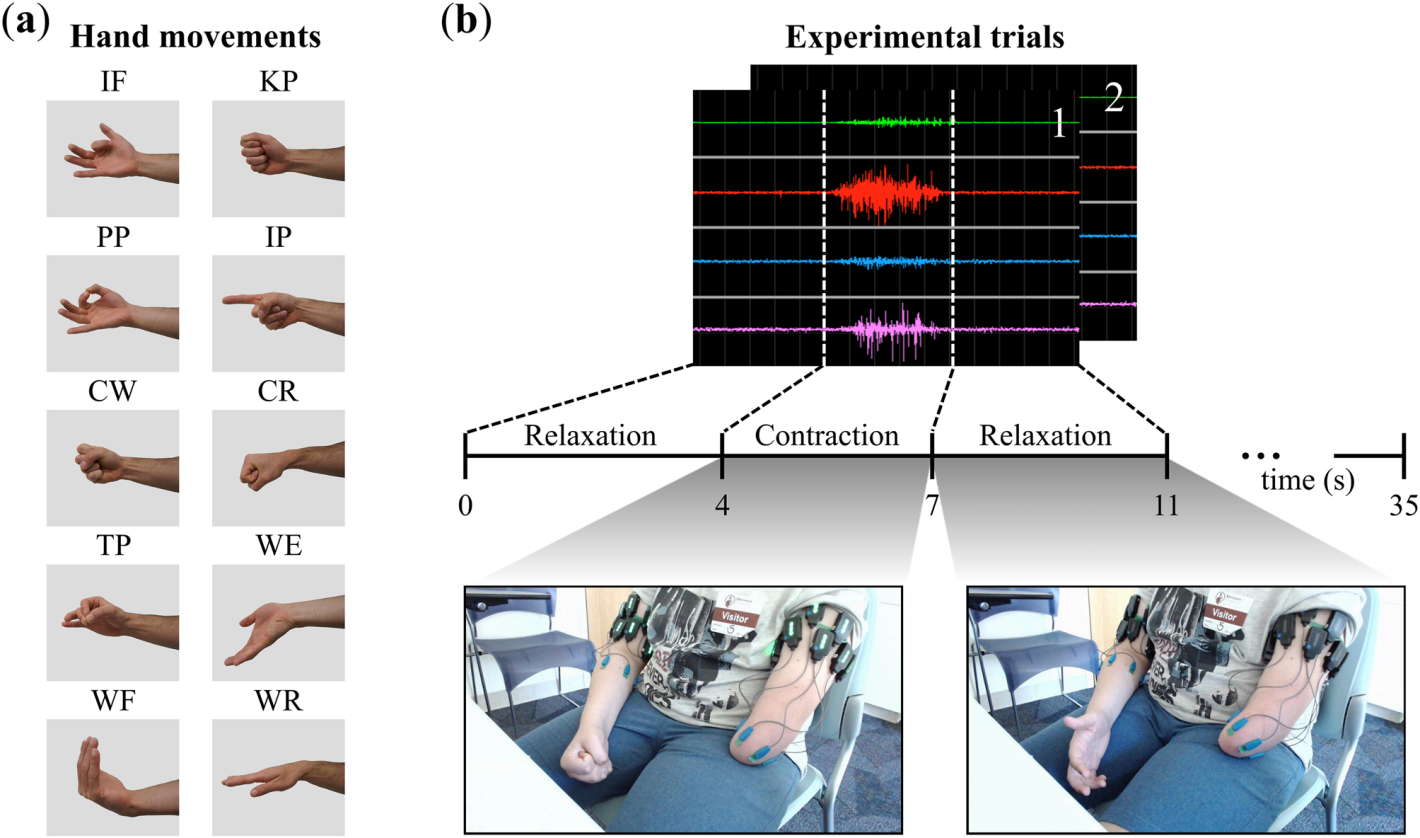
Overview of the experimental outline. (**a**) The 10 hand movements used in the experimental procedures. (**b**) An illustration of the experimental paradigm depicting a trial consisting of relaxation and contraction phases. There were two trials each consisting of alternating phases of four seconds of relaxation and three seconds of contraction for a total of ten contraction and relaxation phases across trials. Participant photo shows a cylindrical warp (CW) contraction and relaxation phase. Figure layout adapted from^39^.

Participants were then informed of which hand movement was to be performed and were allowed to familiarize themselves before proceeding to data collection. A strategic set of 10 hand movements was selected, which included commonly used hand grasps in daily living^25^, individual digit motions, and wrist movements: index flexion (IF), key pinch (KP), pulp pinch (PP), index point (IP), cylindrical wrap (CW), cylindrical wrap wrist rotate (CR), tripod pinch (TP), wrist extension (WE), wrist flexion (WF), and wrist rotation (WR) (Fig. 2a). To ensure rich data sets for analysis of the muscle activity and due to the potential cognitive demands of attempting to move their missing limb, 10 repetitions of each movement were performed. Participants were tasked with envisioning and attempting the prompted hand movement with their affected limb while simultaneously mirroring this action with their unaffected limb. Participants performed the 10 repetitions across 2 trials where a single trial began with the participant in the relaxed phase for 4 s followed by 3 s of contraction as indicated by an auditory tone. The relaxation (no tone) and contraction (tone) phases were repeated 5 times each in a single trial ([5 relaxations and contractions] * 2 trials = 10 relax and contract repetitions for each hand movement). All participants were provided multiple opportunities for rest to mitigate fatigue. The structure of the experimental trials is illustrated in Fig. 2b.

### sEMG pre-processing

A program was written in MATLAB to pre-process (condition) the data collected from the sEMG electrodes by first concatenating data across the 10 repetitions for individual movements. Additionally, to remove the effect of participant reaction times (i.e., from when they first hear the tone to when they contract their muscles), 15% of the data over the relevant time interval (contraction/relaxation) was discarded from both the onset and recession of the movement^26^.

We first performed analysis across sEMG channels, defined as the time-series voltage data produced from an individual sEMG electrode. From each channel we assessed RMS and MNF characteristics, features that are commonly implemented and are sensitive to capturing the biological phenomenon of muscle excitation during hand movements^15,27,28^. For a given limb, movement, and sEMG channel the RMS and MNF were calculated with Eqs. (1) and (2). Where $x_{i}$ is the $i$-th of *N* samples from a single conditioned contraction (3 s) or relaxation (4 s) repetition (e.g. *N* = 6000 Hz * 3 s * 0.7 = 12,600 samples for contraction)^29^. Additionally, $f_{j}$ and $p_{j}$ are the frequency variable and power spectrum, respectively, at a given bin $j$ of *M* frequency bins^29,30^. The frequency range of interest was from 0–1000 Hz, and the bin size was chosen as 0.4 Hz; therefore, the total number of bins was *M* = 2500.

1 $$RMS = \sqrt {\frac{{\mathop \sum \nolimits_{i = 1}^{N} x_{i}^{2} }}{N}}$$

2 $$MNF = \frac{{\mathop \sum \nolimits_{j = 1}^{M} f_{j} p_{j} }}{{\mathop \sum \nolimits_{j = 1}^{M} p_{j} }}$$

For a given limb, the RMS and MNF were normalized to their corresponding maximum across all movements, on an individual channel basis. All data presented here is therefore a percentage of the maximum characteristic per channel.

## Analysis

RMS and MNF characteristics were evaluated in both limbs. This included muscle excitation visualizations, investigating within-movement consistency with Kendall’s Coefficient of Concordance^31,32^ (see below for more details), and performing dissimilarity analysis to understand the distinguishability of hand movements. Furthermore, comparisons of hand movement consistency and the relatedness of movement characteristics across limbs with the RDM condition-label randomization analysis^33^ were conducted. All analyses were performed on an individual participant basis.

### Muscle excitation

#### Visualization

The visualization of muscle excitation across hand movements and limbs was done to observe patterns in muscle activity across sEMG channels for RMS and MNF characteristics. The Shapiro–Wilk Test^34^ was used to assess the normality of the RMS and MNF for each movement on an individual sEMG channel basis. This method was chosen due to its common application with limited sample sizes, as was the case in our study. The majority of the data were found to not be normally distributed. Therefore, all data were plotted using box and whisker plots for each sEMG channel and hand movement which included the relaxation phase.

#### Movement excitation consistency

To understand the degree to which muscle excitation during individual movements were reproducible, measures of consistency were evaluated. We quantified the within-movement consistency given the RMS and MNF muscle excitation across sEMG channels for participants each time the same movement was performed. Due to the nonparametric nature of the RMS and MNF data, the within-movement consistency was determined with Kendall’s Coefficient of Concordance, W^31,32^. This test produces a value of agreement from the ranked order of the 10 repetitions for RMS and MNF characteristics across the number of entities (sEMG channels). Therefore, Kendall’s W provides a measure of agreement (with 0 indicating no agreement and 1 indicating complete agreement) in muscle excitation across sEMG channels (i.e., reproducibility of muscle excitation patterns.) The strength of agreement/consistency adapted from^35–37^ are defined as: W < 0.20 (poor consistency), 0.20 ≤ W < 0.40 (minimal consistency), 0.40 ≤ W < 0.60 (weak consistency), 0.60 ≤ W < 0.80 (moderate consistency), and W ≥ 0.80 (strong consistency).

### Across movement dissimilarity

To examine the distinguishability of muscle response when children with UCBED attempted to perform each hand movement, the spread (given by the interquartile range (IQR)) and median amplitude of both RMS and MNF characteristics were analyzed. In literature, representational geometry is often applied to quantify the distinguishability in measures of physiological activity (e.g., sEMG, EEG, fMRI)^33,38–40^ when participants are presented with different stimuli. Here, we applied and adapted the techniques outlined in^33,38–40^ to assess the distinguishability of muscle excitation (sEMG channels). In this context, we treated the hand movements as the stimuli. Additionally, we illustrated this distinguishability through a qualitative visual representation.

**Figure 3:**
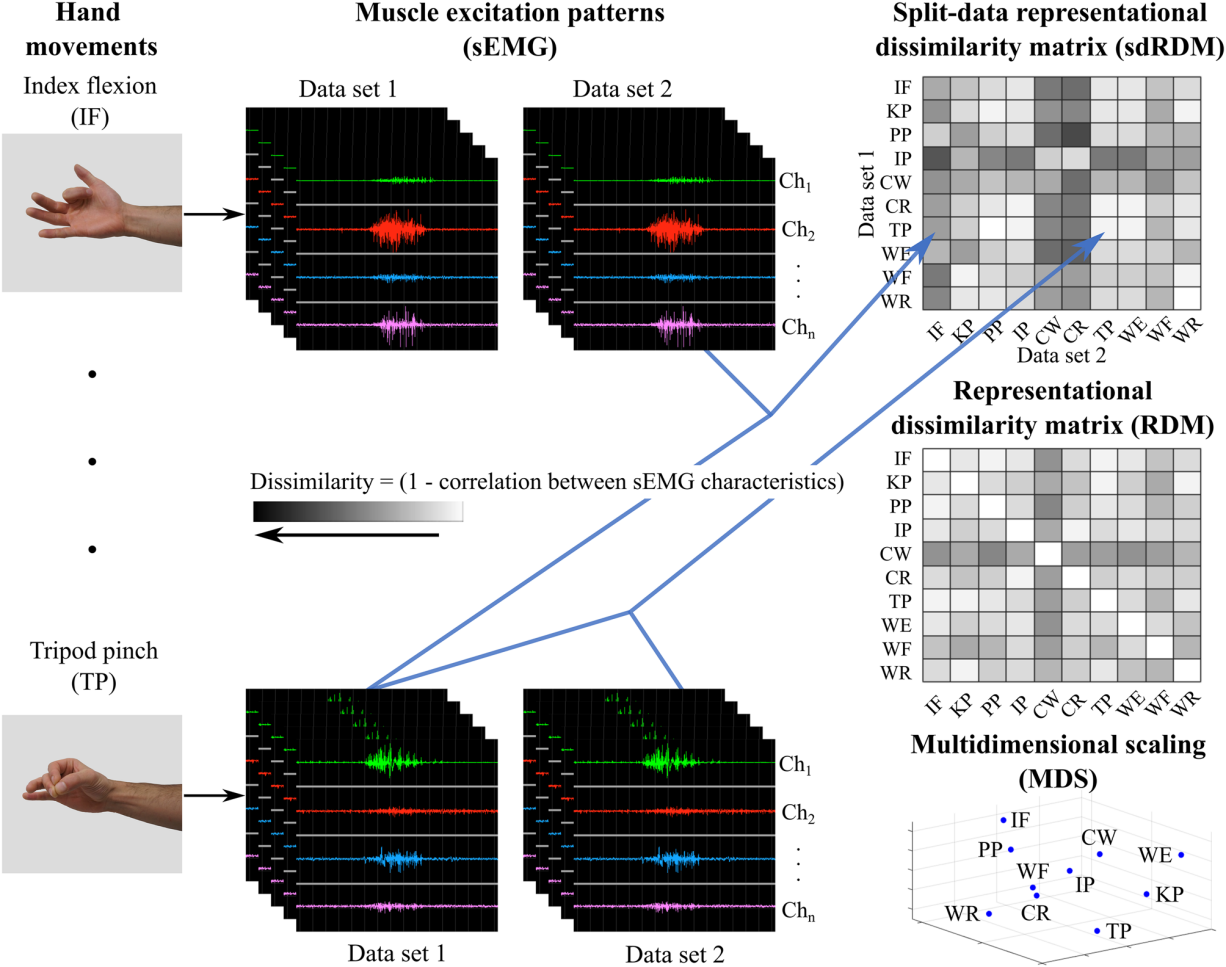
Dataflow to produce the split-data representation dissimilarity matrix (sdRDM) and RDM which are used to understand and visualize distinguishability. Participants attempted various hand movements, and muscle excitation was recorded through sEMG. Data for the sdRDM were split into two data sets containing even and odd trials, and measures of amplitude and spread were calculated from each. Then the sdRDM and RDM were produced with 1 minus the correlation between hand movements for split and unsplit data, respectively. As the dissimilarity increases the color gets darker and as it decreases the color gets lighter. The RDM was produced from unsplit data and used for multidimensional scaling (MDS) visualization and distinguishability across limbs (described further below).

To perform these analyses, a split-data representational dissimilarity matrix (sdRDM) was produced and analyzed. The data generated from performing a hand movement multiple times was first split into two equal data sets containing even and odd repetitions. Then the median and IQR of the RMS and MNF were obtained for each data set on a per-channel basis. Each entry of the sdRDM represented the rank-correlation distance between two data sets for every pair of hand movements across the sEMG channels. This distance was calculated from Kendall’s Tau-b ($\tau_{b}$) rank correlation coefficient, to account for ties, and is defined as (1 − $\tau_{b}$). Kendall’s correlation coefficient was used to accommodate the fact that our data sets were not normally distributed when checked with the Shapiro–Wilk Test^34^. Here the “objects” of Kendall’s correlation coefficient test were defined as the median or IQR of the RMS or MNF for each sEMG channel (depending on which measure was being tested), and the two “variables” were any two pairs of hand movements. Additionally, a representational dissimilarity matrix (RDM) was produced as described above, without splitting the data into two datasets, and multidimensional scaling (MDS) was applied for visualization as adapted from^41^. A depiction of the dissimilarity dataflow as described above is given in Fig. 3.

To determine a distinguishable structure of hand movements across the characteristics, the non-parametric RDM-level condition-label randomization test for exemplar discriminability index (EDI) was performed on the sdRDMs^40^ (hereby referred to as the sdRDM analysis). The EDI was defined as the average between-exemplar dissimilarity estimate (the sdRDM off-diagonals) minus the average within-exemplar dissimilarity estimate (the sdRDM diagonal): EDI = mean(between-exemplar dissimilarity) − mean(within-exemplar dissimilarity), as adapted from^40^. A larger EDI was an indication that the hand movements may be more discriminable, and alternatively, smaller EDI values suggested hand movements are less discriminable. The sdRDM analysis consisted of simulating the null distribution by performing an exhaustive permutation of the rows of the sdRDM and calculating the EDI for every permutation. Therefore, we define the null hypothesis H_0_ as there being no structure to the data and pairwise distances of hand movements being equal (i.e., the intra-movement and inter-movement dissimilarities can be shuffled without change to the sdRDM structure). The EDI of the unshuffled sdRDM was then calculated and used to estimate the *p*-value by a proportion, i.e., by taking the EDIs in the null distribution that were greater than the unshuffled EDI. A significance level of α = 0.05 was used. A *p*-value less than this threshold would indicate that there is significant structure in the data and that pairwise distances of hand movements are not equal. This would suggest that as participants performed missing hand movements the muscle excitation derived from these motions were distinguishable from one another.

To qualitatively visualize the distinguishability across hand movements, the RDM constructed from both data sets (i.e., unsplit data) was used and multidimensional scaling (MDS) was applied (Fig. 3). MDS is a dimensionality reduction technique with the objective of reflecting the dissimilarity between items by projecting their distances in a lower dimensional space^38^. Nonmetric MDS was applied to the dissimilarity matrices (RDMs) through the MATLAB function *mdscale* with the squared stress criterion (*sstress*)^39^. The *statset* parameter for the maximum number of iterations (*MaxIter*) was increased to allow for convergence of the MDS solution.

### Differences across limbs

#### Consistency across limbs

To further understand if the consistency of the children’s affected muscle excitation was similar to that of their unaffected limb, across-limb comparisons were made. The previously-discussed within-movement consistency (across all hand movements), was used to determine overall consistency for each limb. Therefore, all movement consistencies for the affected limb were compared to all movement consistencies for the unaffected limb of a given participant. First, we assessed the normality for measures of consistency across the 10 movements for each limb with the Shapiro–Wilk Test^34^. Then, due to the non-parametric nature of the data, a Wilcoxon Signed Rank test with an α = 0.05 was used for comparisons. The null hypothesis, H_0_, was defined such that the difference between the median movement consistency across the two limbs is zero. In this way, we were able to determine if there were any statistical differences in the participants’ ability to attempt repeatable missing hand movements with their affected limb compared to their unaffected limb.

#### Relatedness across limbs

In order to understand if attempted hand movements were related across the affected and unaffected limbs, measures of the RMS and MNF characteristics were evaluated with the non-parametric RDM label randomization analysis adapted from^33^. The affected limb RDM was selected, and 50,000 random permutations of both the rows and columns were performed. At each permutation, the RDM of the affected limb and the unaffected limb were compared with Kendall’s Tau-a ($\tau_{a}$) correlation, provided in the representational similarity analysis toolbox^42^, thereby allowing for simulation of the null distribution. The null hypothesis, H_0_, is defined as two RDMs being unrelated. We calculated the correlation between two RDMs, which were subject to no label permutations, and found an estimate for the *p*-value through the proportion of correlations in the null distribution greater than the non-permuted correlation^33^. A significance level of α = 0.05 was selected and if the estimated *p*-value was less than the significance level then there was favor for the alternative hypothesis H_1_ that there is relatedness between the two RDMs. This suggests that the muscle excitation produced when participants performed missing hand movements is statistically related to that of their unaffected limb.

## Results

### Muscle excitation

#### Visualization

**Figure 4:**
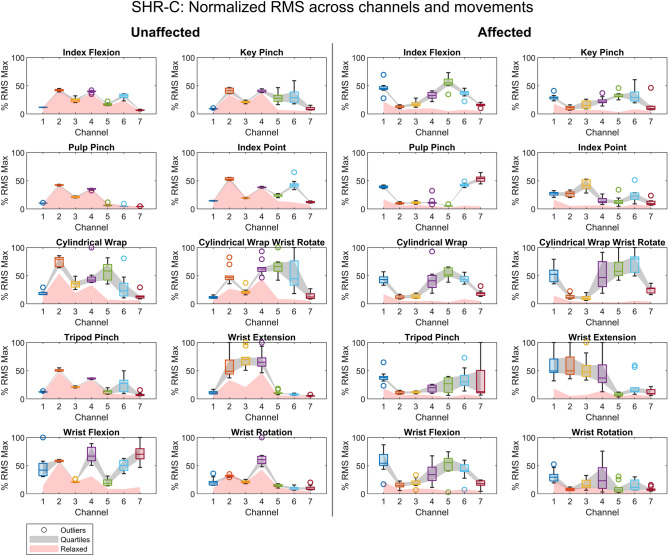
The box and whisker plots provide a visualization of the RMS muscle excitation patterns and relaxed states seen for the various hand movements across the unaffected and affected limbs for participant SHR-C.

**Figure 5:**
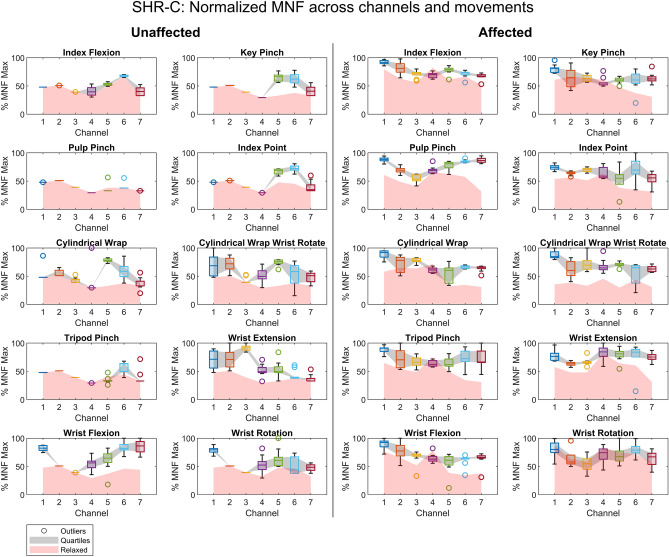
The box and whisker plots provide a visualization of the MNF muscle excitation patterns and relaxed states seen for the various hand movements across the unaffected and affected limbs for participant SHR-C.

To visualize sEMG patterns across channels, the median RMS and MNF muscle excitation across limbs and hand movements were calculated as participants attempted to perform each motion (Figs. 4, 5, and Supplementary Figs. S1–S18 online). For the RMS characteristic, the unaffected limb exhibited visual signs of normal muscle function. That is, as participants performed various movements, only a subset of sEMG channels recorded muscle excitation. This subset varied based on the attempted movement, indicating coordinated patterns of muscle excitation were being enacted, which is considered normal in unaffected healthy limbs^43^. For example, during wrist extension and flexion, the extensor carpi radialis longus and flexor carpi radialis muscles are activated separately for each wrist movement^43^. A similar phenomenon can be seen in Fig. 4 as exemplified by participant SHR-C and throughout participant data sets (Supplementary Figs. S1–S18 online). Different patterns of sEMG activity were observed for the RMS characteristic across attempted hand movements of participants' affected limbs, thereby providing a visual indication of the extent to which children with UCBED could actuate their affected muscles. Interestingly, although not in all cases, when the participants were asked to attempt a hand movement with their affected limb the majority of sEMG channels recorded muscle excitation above the relaxed state (see Fig. 4 and the RMS Supplementary Figures online). Additionally, it is important to note that for some participants the affected limb RMS characteristic across the sEMG channels demonstrated relatively large degrees of variability, e.g., participant SHR-B produced a large RMS spread across sEMG channels for most hand movements (see Supplementary Fig. S3 online). In contrast, participant SHR-D had very few visual patterns of RMS muscle excitation with similar spread across movements. Therefore, we observed varied visual sEMG patterns of RMS muscle excitation across hand movements and participants. This visual inspection was crucial prior to performing statistical evaluations, particularly for identifying patterns within the sEMG voltage data. It played a key role in guiding the subsequent statistical analyses conducted in the proceeding sections.

For the MNF characteristic, patterns were illustrated in frequency-based muscle excitation for the unaffected and affected limbs among participants across sEMG channels. The MNF muscle excitation for both limbs showed parallels to their RMS counterparts. While the unaffected excitation differed across sEMG channels from movement to movement, the majority of the affected excitation was above the relaxed state, as illustrated by participant SHR-C in Fig. 5 and the MNF Supplementary Figures online. Although there were variations in the degree of visual patterns for the RMS characteristics across movements, the MNF characteristics revealed additional patterns for most participants across both limbs (see MNF Supplementary Figures online). This indicates that separately and together RMS and MNF characteristics may provide sufficient information to differentiate between hand movements.

#### Movement excitation consistency

**Figure 6:**
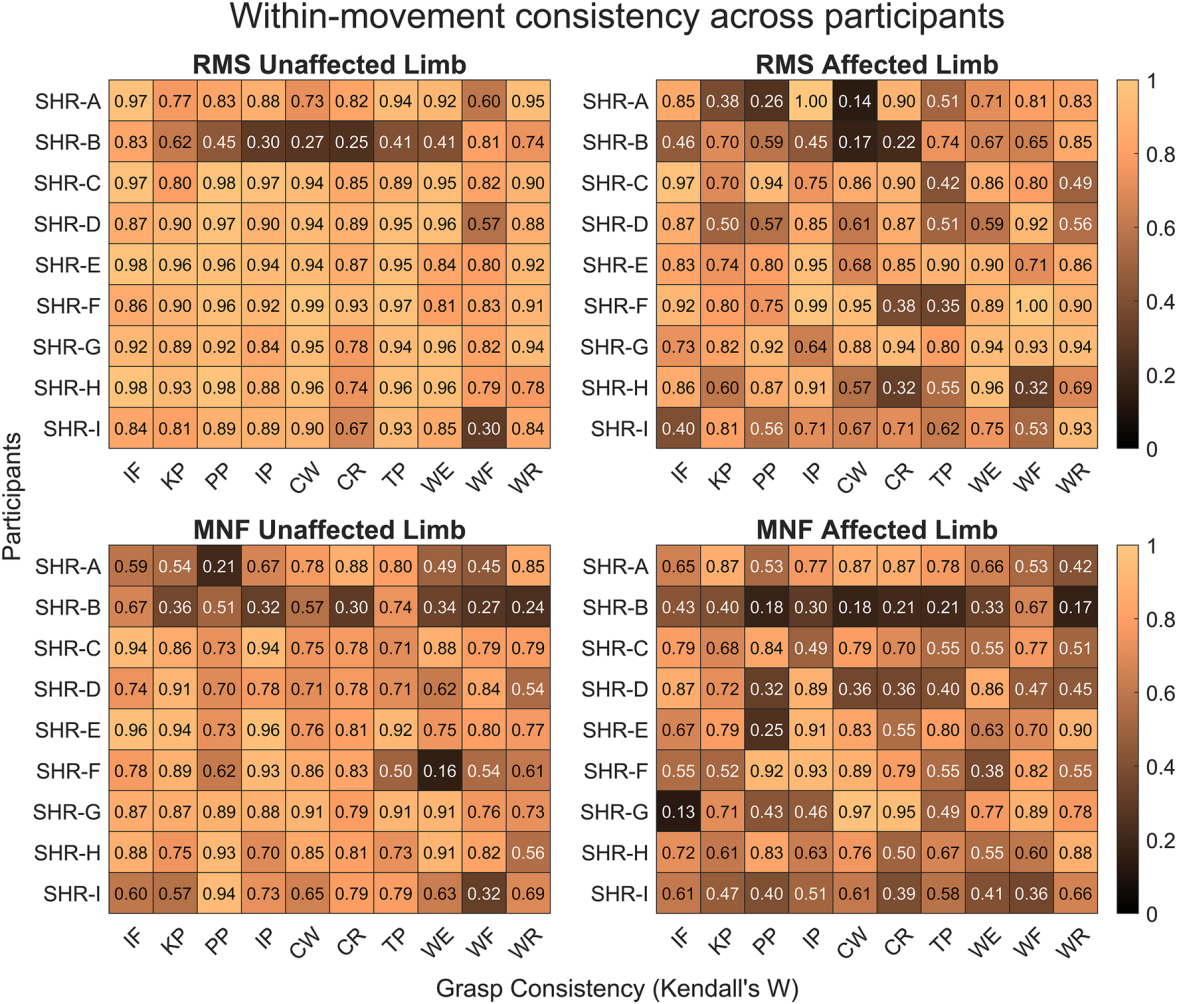
Within-movement consistency for median RMS and MNF data across repetitions obtained from Kendall’s Coefficient of Concordance, W. As the color gets darker this indicates a decrease in within-movement consistency, and the lighter the color an increase in consistency. The ranges of consistency are defined as W < 0.20 (poor consistency), 0.20 ≤ W < 0.40 (minimal consistency), 0.40 ≤ W < 0.60 (weak consistency), 0.60 ≤ W < 0.80 (moderate consistency), and W ≥ 0.80 (strong consistency).

Within-movement consistency was calculated from Kendall’s Coefficient of Concordance, W, to quantify participants' ability to consistently perform patterns of RMS and MNF muscle excitation for each limb over the multiple trials of hand movements. Here consistencies ranged from W < 0.20, 0.20 ≤ W < 0.40, 0.40 ≤ W < 0.60, 0.60 ≤ W < 0.80, and W ≥ 0.80 with poor, minimal, weak, moderate, and strong consistency, respectively. The definition of consistency in this work was adapted from^35–37^ and represents a novel measure not previously applied in this context. These values were utilized to demonstrate consistency and assess the affected limb in reference to the healthy, unaffected limb as a control. In general, for the unaffected limb, measures of consistency ranged from moderate to strong (0.60 ≤ W ≤ 0.99), providing a baseline for healthy, typical, repetitive muscle actuation; few participants had poor to weak consistency (0.16 ≤ W ≤ 0.59). Specifically, for the RMS consistency, all participants except for SHR-B had at least 9 hand movements with moderate to strong consistency i.e., a W of greater than 0.60. Interestingly, SHR-B had the top two lowest RMS consistencies in the unaffected limb for the movement-type cylindrical wrap with wrist rotated (CR) and cylindrical wrap (CW), with values of W = 0.25 and W = 0.27, respectively. SHR-B only had 4 out of 10 hand movements with moderate to strong consistencies. Similarly, participant SHR-I had the third lowest consistency of W = 0.30 for wrist flexion (WF). Alternatively, for the MNF unaffected limb within-movement consistency, all participants except SHR-B had moderate to strong consistency values for at least 5 hand movements while SHR-B had only 2. The top three lowest consistencies were present in participants SHR-F (W = 0.16 for wrist extension (WE)), SHR-A (W = 0.21 for pulp pinch (PP)), and SHR-B (W = 0.24 for wrist rotation (WR)) in the poor to minimal consistency range. All hand movement consistency values for the unaffected limb can be seen in the first column of Fig. 6.

When attempting to move their missing limb, participants were able to produce repeatable patterns of affected muscle excitation, although there was variability across participants. Repeatable patterns are an important facet for robust control of prostheses given that 6–9 common hand movements can account for nearly 80% of activities in daily living^25,44^, indicating the efficacy of prosthesis use in this population. When evaluating the RMS within-movement consistency in the affected limb, we found at least 5 of the 10 hand movements had moderate to strong consistency. Here, participant SHR-A had the lowest consistency value of W = 0.14 for the cylindrical wrap (CW). Participant SHR-B had the second and third lowest consistency values: W = 0.17 in the cylindrical wrap (CW) and W = 0.22 in the cylindrical wrap with wrist rotated (CR). Additionally, the MNF within-movement consistency for the affected limb showed that all participants except SHR-B had moderate to strong consistency values for 4 or more hand movements. SHR-G had the lowest consistency value of W = 0.13 for index flexion (IF). Participant SHR-B had only 1 hand movement, wrist flexion (WF), with a moderate consistency value, and had the second through the sixth lowest consistency values from poor to weak. All other consistency values for the affected limb can be seen in the second column of Fig. 6. Together, we see that 5 of 10 movements for RMS and 4 of 10 movements for MNF approached moderate to strong consistency (i.e., nearing the consistency for 6–9 hand movements). In conclusion, this may indicate that participants were able to perform reproducible attempted hand movements in their affected limb with a consistency range similar to that of their healthy unaffected limb (0.60 ≤ W ≤ 0.99). Statistical comparisons of consistency measures are further investigated in “Consistency across limbs” Section to gain a deeper understanding of the differences across limbs.

### Across movement dissimilarity

To further understand whether the hand movements were dissimilar or distinguishable from one another, sdRDM analysis was performed. This assessment was motivated given the emergence of different muscle excitation patterns observed across hand movements in “Visualization” Section. The objective was to determine whether there was sufficient information for distinguishable structure across hand movements within the measures of RMS and MNF characteristics. The investigation of the median RMS and MNF enabled us to understand whether the central tendency of the hand movement muscle excitation exhibited distinguishable structure. Additionally, assessing the spread/variability (i.e., interquartile range (IQR)) of these characteristics allowed us to determine whether the reliability of hand movement muscle excitation also exhibited a distinguishable structure. Collectively, this analysis provided an indication of typical muscle excitation by assessing the distinguishability between movements for measures of RMS and MNF. It is noteworthy that typical muscle excitation involves the variability in sEMG signals across channels from task to task, or even within the same movement^15^. Furthermore, we employed multidimensional scaling (MDS) to qualitatively visualize the distinguishability across hand movements.

The sdRDM analysis of the median and IQR measures for RMS and MNF indicated that distinguishable structures of sEMG data were present when the participants with UCBED attempted to perform missing hand movements. This analysis also showed distinguishable structures of sEMG data were present in participants’ unaffected limbs, as is expected of typical muscle contractions. However, this was not the case for all participants e.g., the analysis did not provide sufficient evidence to suggest there was a distinguishable structure for the median RMS of SHR-B’s unaffected and affected limbs with *p* = 0.188 and *p* = 0.267, respectively. Additionally, sdRDM analysis of the affected limb did not provide sufficient evidence to suggest a distinguishable structure for the following participants: SHR-D (*p* = 0.106, RMS IQR), SHR-F (*p* = 0.118, MNF IQR), and SHR-I (*p* = 0.163, RMS IQR). All other sdRDM analyses for the affected and unaffected limb showed a distinguishable structure of the hand movements; all *p*-values for each limb across participants and characteristics can be found in Table 2.

**Table 2:** Split-data representational dissimilarity analysis to distinguish the structure of amplitude and spread of RMS and MNF characteristics.

Participants	Unaffected				Affected			
	RMS EDI		MNF EDI		RMS EDI		MNF EDI	
	Median	IQR	Median	IQR	Median	IQR	Median	IQR
SHR-A	0.552 *p* < 0.001	0.483 *p* < 0.001	0.629 *p* < 0.001	0.235 *p* = 0.005	0.319 *p* = 0.003	0.459 *p* = 0.006	0.519 *p* < 0.001	0.452 *p* < 0.001
SHR-B	0.095‡	0.546 *p* < 0.001	0.252‡	**0.728†**	0.047‡	0.256 *p* = 0.036	0.167‡	**0.510†**
	*p* = 0.188*		*p* = 0.018	*p* < 0.001	*p* = 0.267*		*p* = 0.023	*p* < 0.001
SHR-C	0.550 *p* < 0.001	0.472 *p* < 0.001	0.720 *p* < 0.001	0.377 *p* < 0.001	0.535 *p* < 0.001	0.294 *p* = 0.003	0.571 *p* < 0.001	0.423 *p* < 0.001
SHR-D	0.455 *p* < 0.001	0.463 *p* < 0.001	0.667 *p* < 0.001	0.205‡	0.466 *p* < 0.001	0.114‡	**0.665†**	0.199 *p* = 0.015
				*p* = 0.020		*p* = 0.106*	*p* < 0.001	
SHR-E	0.421 *p* < 0.001	0.453 *p* < 0.001	0.648 *p* < 0.001	0.235 *p* = 0.007	0.489 *p* < 0.001	0.273 *p* < 0.001	0.599 *p* < 0.001	0.212 *p* = 0.018
SHR-F	0.554 *p* < 0.001	0.459 *p* < 0.001	0.438 *p* < 0.001	0.603 *p* < 0.001	0.474 *p* < 0.001	0.254 *p* = 0.004	0.590 *p* < 0.001	0.119‡
								*p* = 0.118*
SHR-G	**0.635†**	**0.578†**	**0.783†**	0.449 p < 0.001	**0.629†**	**0.519†**	0.529 *p* < 0.001	0.303 *p* = 0.007
	*p* < 0.001	*p* < 0.001	*p* < 0.001		*p* < 0.001	*p* = 0.002		
SHR-H	0.457 *p* < 0.001	0.368‡	0.468 *p* < 0.001	0.214 *p* = 0.007	0.351 *p* < 0.001	0.212 *p* = 0.008	0.233 *p* = 0.005	0.364 *p* < 0.001
		*p* < 0.001						
SHR-I	0.552 *p* < 0.001	0.497 *p* < 0.001	0.453 *p* < 0.001	0.366 *p* < 0.001	0.474 *p* < 0.001	0.116	0.309 *p* < 0.001	0.180 *p* = 0.021
						*p* = 0.163*		

*Italic *p*-values from the sdRDM analysis indicate a failure to reject the null hypothesis (i.e., there was not sufficient evidence to suggest a distinguishable structure of the hand movements), given a significance level of α = 0.05.

^†^Bold EDI values indicate the maximum within a column.

^‡^Underline EDI values indicate the minimum within a column.

Further investigation of the sdRDM exemplar discriminability index (EDI) highlighted participants that may be good candidates for control of dexterous prostheses i.e., large amplitude-based (median) EDI values suggest increased distinguishability of prompted missing hand movements. Additionally, the large EDI values for IQR measures indicated two findings. First, typical muscle excitation, which is indicative of distinguishable movements and therefore potentially effective control of dexterous prostheses. Second, variability may be too large across sEMG signals which may indicate poor consistency, upon which the muscle excitation plots and measures of consistency should be further investigated. Here, maximum and minimum EDI values are highlighted within the RMS or MNF measures for the unaffected and affected limbs across participants. SHR-G had the majority of maximum EDI values for 3 of the 4 measures of the unaffected limb and 2 of the 4 measures for the affected limb. In contrast, SHR-B had the majority of minimum EDI values for 2 of the 4 measures of the unaffected and affected limb, respectively. EDI values across limbs for measures of RMS and MNF are highlighted for each participant in Table 2.

**Figure 7:**
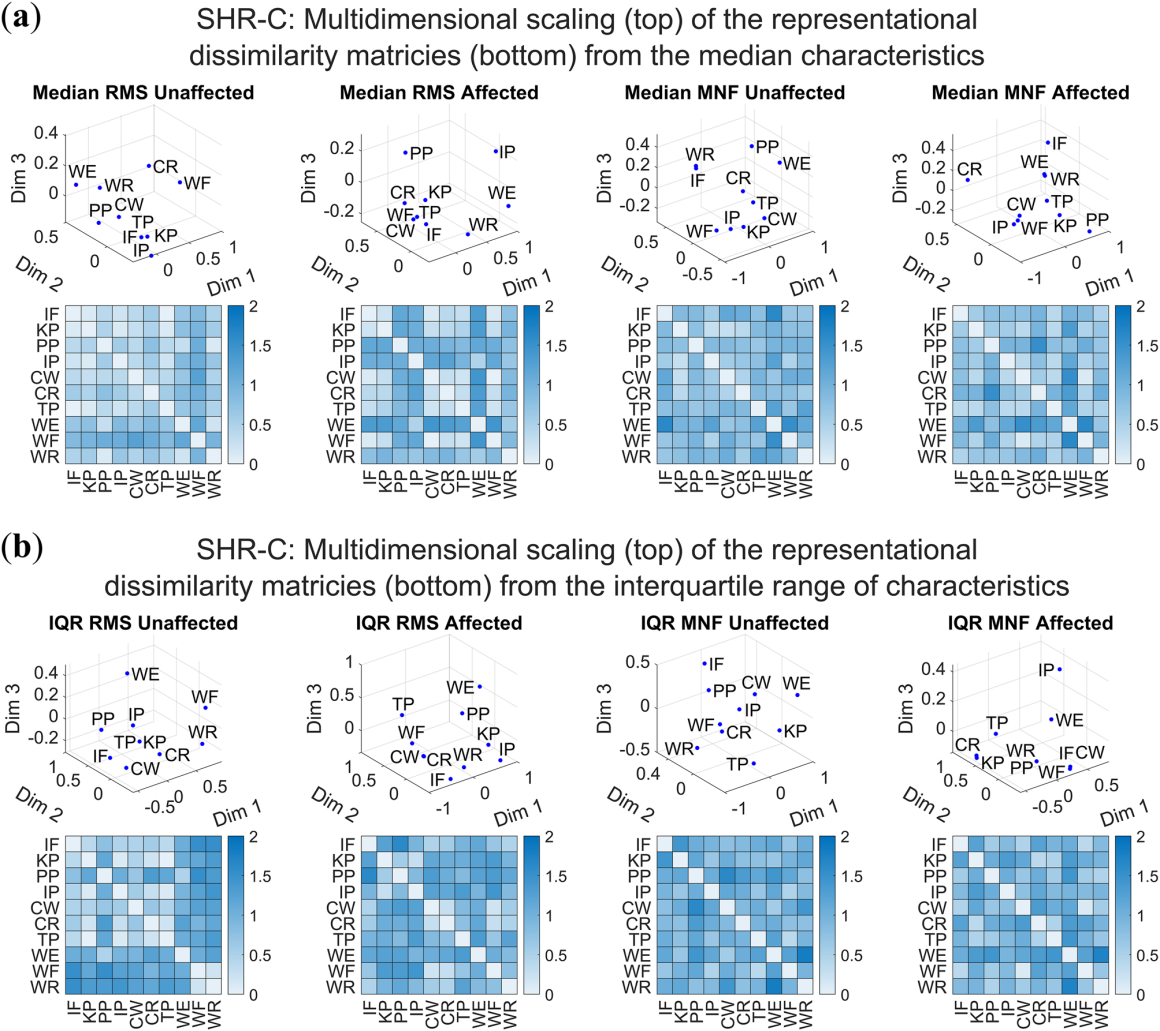
Visual representation of the correlation distances between hand movements for the amplitude and spread of measurement characteristics of SHR-C. The multidimensional scaling plots are provided in three dimensions corresponding to the representational dissimilarity matrices for (**a**) the median RMS and MNF characteristics and (**b**) the RMS and MNF interquartile range (IQR).

To qualitatively visualize the differences across hand movements, the complete data set across trials (unsplit) was used to create the RDM for each limb. As previously mentioned, nonmetric MDS was used on each limb’s RDM to reflect the higher dimensional distances across hand movements in a three-dimensional subspace. The MDS illustrated distinguishable differences between the various hand movements with few motions close and/or overlapping in the subspace as seen by the separation of points in the top panel of Fig. 7a,b. Figure 7 shows both the median and IQR of RMS and MNF characteristics with the MDS (top) and corresponding RDM (bottom). Additionally, all MDS and RDM plots for participants can be seen in Supplementary Figs. S19–S27 online. These results reveal, through a qualitative visualization, that the measures analyzed provide information to distinguish attempted hand movements by visual separation.

### Differences across limbs

#### Consistency across limbs

**Figure 8:**
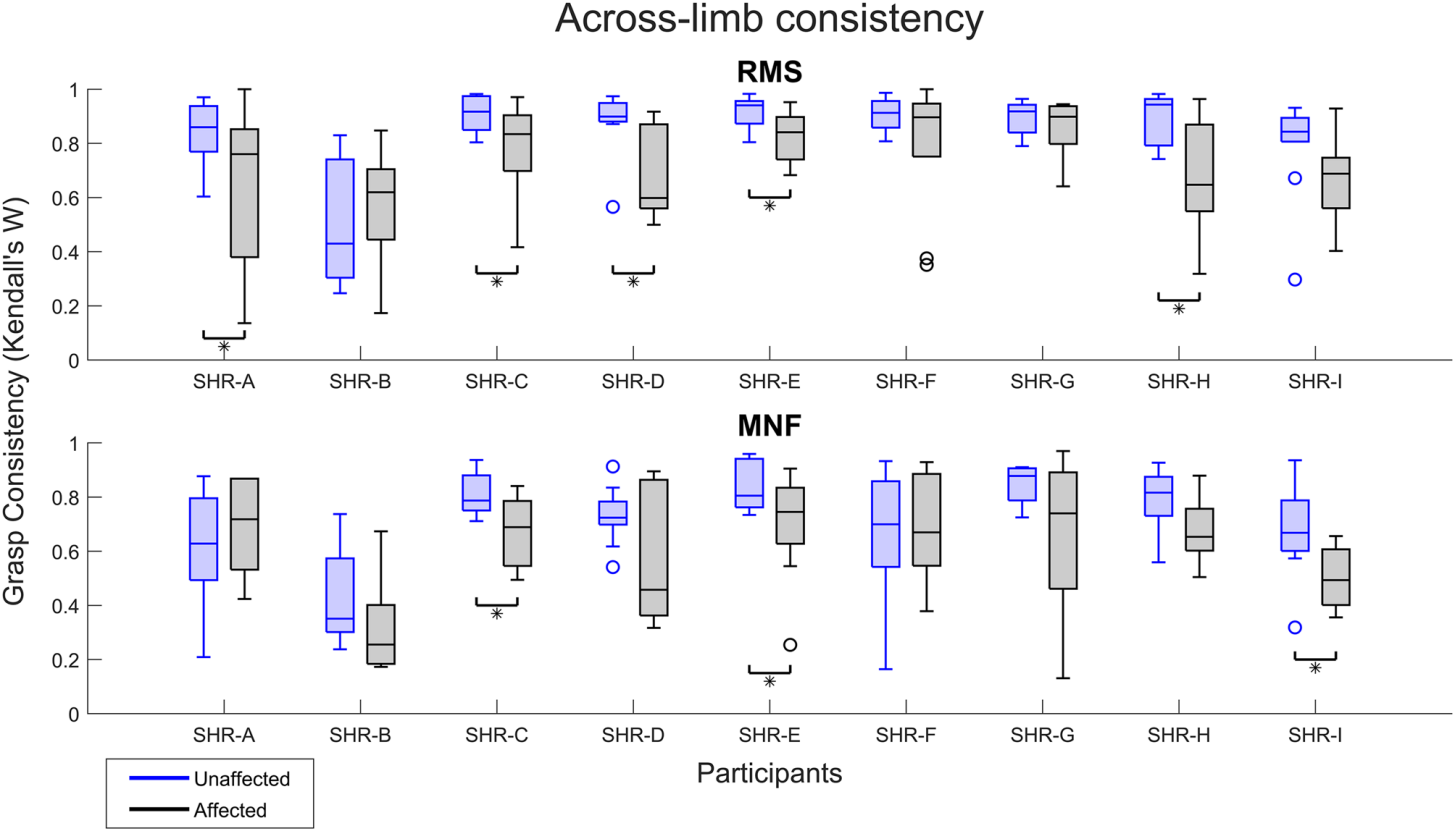
Comparison of across-limb consistency for the 10 hand movements. Participant comparisons are done with a Wilcoxon Signed Rank test given a significance level of α = 0.05. Blue shading refers to the unaffected limb while black shading is for the affected limb. *Represents those participants where the across-limb consistency was found to be in favor of the alternative hypothesis i.e., the difference in the median consistency across the two limbs was not zero.

To get a better understanding of the differences in the participants’ ability to attempt consistent hand movements with their affected limb, statistical comparisons were made to the consistency of their unaffected limb. We found RMS consistency in the affected limb to be statistically lower than the unaffected limb for participants SHR-A, C, D, E, and H. Alternatively for MNF, participants SHR-C, SHR-E, and SHR-I had statistically lower consistency in the affected limb when compared to the unaffected limb. In each of the preceding cases, the unaffected limb had an overall higher median than the affected limb. The consistency for RMS and MNF across participants with highlighted statistical differences are shown in the top and bottom panels of Fig. 8, respectively. The resulting consistencies in RMS and MNF across limbs (Fig. 8) indicated that some participants had difficulty reproducing hand movements to a similar degree as that of their unaffected limb. This inability to reproduce movements, in turn, may hinder their potential to use multi-grasp prostheses.

#### Relatedness across limbs

To understand if the structure of the affected limb RDM was related to that of the unaffected limb, the non-parametric RDM label randomization test was used^33^. It was found that the majority of participants had some degree of shared information such that the pair-wise distances between hand movements for their affected limb was related to that of their unaffected limb. There was relatedness with the exception of the following participants: SHR-F (*p* = 0.060, RMS IQR), SHR-B (*p* = 0.055, median MNF), and SHR-F (*p* = 0.054, median MNF). Additionally, Table 3 shows the non-permuted Kendall’s Tau-a ($\tau_{a}$) correlations used for the RDM test with corresponding *p*-values. These results indicate that the majority of participants had related information across limbs for amplitude and spread of RMS and MNF characteristics.

**Table 3:** Relatedness of RDMs across limbs for amplitude and spread of RMS and MNF characteristics.

Participants	RDM relatedness across limbs			
	RMS correlation		MNF correlation	
	Median	IQR	Median	IQR
SHR-A	0.219 *p* = 0.002	0.266 *p* = 0.001	0.234 *p* = 0.002	0.288 *p* < 0.001
SHR-B	0.401 *p* < 0.001	0.182 *p* = 0.015	*0.119* *p* = 0.055*	0.381 *p* < 0.001
SHR-C	0.255 *p* = 0.005	0.159 *p* = 0.017	0.318 *p* < 0.001	0.293 *p* < 0.001
SHR-D	0.272 *p* < 0.001	0.215 *p* = 0.005	0.180 *p* = 0.008	0.364 *p* = 0.001
SHR-E	0.465 *p* < 0.001	0.262 *p* < 0.001	0.298 *p* = 0.001	0.254 *p* < 0.001
SHR-F	0.200 *p* = 0.005	*0.116* *p* = 0.060*	*0.123* *p* = 0.054*	0.200 *p* = 0.003
SHR-G	0.439 *p* < 0.001	0.349 *p* < 0.001	0.204 *p* = 0.002	0.301 *p* < 0.001
SHR-H	0.224 *p* = 0.014	0.158 *p* = 0.013	0.423 *p* < 0.001	0.200 *p* = 0.005
SHR-I	0.179 *p* = 0.007	0.144 *p* = 0.030	0.202 *p* = 0.011	0.137 *p* = 0.023

*Italic cells highlight values from the RDM label randomization test, which indicate there was not sufficient evidence for the relatedness across limbs (with a significance level of α = 0.05).

## Discussion

This study investigated the extent to which affected muscles respond when children with UCBED attempted to perform various hand movements. We have shown that even though these children were born with limb deficiency and have never used their affected muscles to actuate an intact limb there is still patterned, consistent, and distinguishable muscle activity in response to attempted missing hand movements.

### Visible patterns of muscle excitation

Across most participants, different sEMG patterns of muscle excitation for both RMS and MNF characteristics were seen in the affected limb indicating their ability to excite coordinated muscle excitation as they attempted the various hand movements (Figs. 4, 5, and Supplementary Figs. S1–S18 online). Furthermore, as expected in the unaffected limb, there were differences in muscle excitation as captured across sEMG channels. Although this was true for many participants’ affected limbs, it was not true for all. For example, SHR-A had few visual patterns in their RMS muscle excitation across attempted hand movements, which can be seen in the Supplementary Fig. S1 online. This finding is likely attributed to the affected limb circumference (15 cm) and length (13 cm), the smallest circumference out of the entire cohort. Therefore, due to the size, it is doubtful that the participant has developed similar muscular structure and mass to those participants with longer affected limbs. Additionally, participant SHR-B showed a large interquartile range in RMS muscle excitation in their affected limb and interestingly, similar results were also observed in their unaffected limb. This large spread in data across the hand movements and limbs was likely attributed to the child’s age, as they were the youngest (8 years old) among our participants; their short attention span for the experimental task thereby affected movement reproducibility. Finally, when viewing participant SHR-D there was apparent muscle excitation, although few visual patterns emerged in the affected limb for both RMS and MNF characteristics across the attempted hand movements. Although we believe that the limited observed patterns and large spread across data may have been attributed to a premature sensory-motor system in participants SHR-B and SHR-D, given their ages (8 and 9 years old, respectively) and the fact that their limbs have not fully developed, further investigation is required. This would involve larger cohorts spanning various ages and limb lengths to quantify these effects. Though it is clear that most participants can generate coordinated muscle activation in their affected side, it also appears that limb size may be a relevant factor in detecting muscle activity. Furthermore, age and cognitive factors such as attention spans may also be important in the reproducibility of muscle excitation and ultimately the efficacy of advanced prosthesis control systems.

### Reproducible attempted hand movements

The participants' ability to reproduce attempted hand movements was explored through measures of within-movement consistency. The unaffected limb exhibited quantitatively higher consistency than that of the affected limb. However, when comparing the median consistency across limbs for each participant, only a few measures were found to be significantly different (Fig. 8). One exception was participant SHR-B who had the lowest consistency across both limbs for RMS and MNF characteristics, which aligns with the visualization of muscle excitation that depicted large interquartile ranges across sEMG channels (see Supplementary Figs. S3 and S4 online). Additionally, the MNF consistency was quantitatively lower than that of the RMS across participants. This finding may be attributed to MNF itself because although it is a robust measure of muscle excitation, it is often used as an assessment of and is sensitive to muscle fatigue^15,28^. Even though participants were given multiple rest periods, it is possible they became fatigued at various stages which would explain our findings. Fatigue may be particularly relevant in this cohort given that despite consistent patterns of movement-to-movement muscle excitation, the activity may be deemed as physically demanding because they have never contracted their affected muscles in these repeatable, distinct ways. However, MNF remains a relevant measure for investigating muscle excitation in this unique population, as it is commonly employed when assessing alternative measures of physiological muscle activity^28^. Additionally, participants’ ability to produce repeatable patterns of muscle excitation in their affected limb suggests the potential for the use of multi-grasp prostheses. From this, it follows that given proper training, consistency is expected to approach that of their unaffected limb (i.e., nearing the consistency for 6–9 hand movements). Aside from training, an investigation into optimal sEMG characteristics, apart from RMS and MNF, should be explored for device control. The effects of physical conditioning, training, fatigue, and optimal sEMG characteristics are all important considerations for the use of advanced prostheses and muscle-based control systems in this population.

### Distinguishable attempted hand movements

There was a distinguishable structure to the RMS and MNF characteristics in the affected limb for the majority of the participants (Table 2). Moreover, there was a degree of relatedness across limbs as children attempted missing hand movements (Table 3). However, this was not true for all participants. For example, participant SHR-B did not show a distinguishable structure for median RMS and relatedness for the median MNF across limbs, as previously shown in the results. This may support the large spread seen in the muscle excitation across sEMG channels and the lack of consistency in hand movements for this individual participant (see Supplementary Figs. S3 and S4 online). As discussed previously, these findings are likely attributed to factors of age and limb size. For measures of spread, some participants did not demonstrate a distinguishable structure (i.e., SHR-D and SHR-I for RMS IQR, and SHR-F for MNF IQR). Moreover, for the latter (SHR-F), we did not find that the pair-wise distances between hand movements for the RMS IQR measures were related across limbs. We found muscle excitation was conceivably both distinguishable across movements and related across limbs as a result of participants simultaneously mirroring hand movements. Accordingly, participants were trying to imagine performing the same movement to the same degree with every repetition. As a result, this still strongly indicates children’s potential ability to actuate their affected muscles in distinguishable and consistent patterns, and with proper training may be able to effectively control dexterous prostheses.

The EDI values (Table 2) provided additional insight into the distinguishability of the muscle excitation structure. To note, participant SHR-G had the majority of maximum EDI values across all participants and limbs. This was likely attributed to a more mature sensory-motor system as the participant was 19 years old and uses a myoelectric prosthesis; that is, they control their device using the sEMG of their affected muscles. Although not a pattern recognition sEMG control system that necessitated contraction of their affected muscles in unique patterns, this participant still used 2-site control that required isolation and contraction of larger muscle groups in their affected limb. Presumably, this may have ameliorated their ability to generate consistent distinguishable muscle excitation patterns when prompted during the experiment. Participant SHR-B had a majority of the lowest EDI values across limbs, which parallels the previously discussed consistency and distinguishability results. Undoubtedly, these two participants can allow us to begin understanding the variability that may be present in this population of children and also provides a further appreciation for the improvements possible with regular prosthesis use and actuation of affected muscles through training.

The multidimensional scaling (MDS) plots are useful to visualize the distinguishability of the various hand movements. Although most participants showed a distinguishable structure across movements (see Supplementary Figs. S19–S27 online), there were a few exceptions. The RMS and MNF correlation distances between hand movements were determined by the median across repetitions. If a participant had low movement consistency the median would not be useful to define the motion, and the RDM and MDS plots would not demonstrate the distinguishability of hand movements. We can see this clearly demonstrated in participant SHR-B, who exhibited poor RMS and MNF consistency measures of muscle excitation for both the affected and unaffected limb.

## Conclusions

We have shown in a limited cohort of children with UCBED that they possess a degree of biological control over their affected muscles with the ability to perform consistent and distinguishable hand movements. However, not all participants were able to achieve this degree of biological control over their affected muscles which may have been attributed to age and limb size, since cognitive demands and robust musculature are important to achieve meaningful muscle activation. A limitation of this study that could have affected the results was that we did not have a cohort of age- and sex-matched able-bodied participants to serve as a ground truth control for typical muscle excitation. Nevertheless, the participants underwent typical development and maturation, aside from their affected limb, allowing their unaffected limb to serve as an internal control for comparison. Our findings also suggest that children with UCBED may have the ability to use advanced prosthesis control systems. However, further work is needed to examine age and limb size with larger cohorts and more statistical power to effectively adapt, translate, and prescribe dexterous prostheses to the pediatric population. The bulk of literature for dexterous upper limb prosthetic devices is saturated in adult-based control systems using sEMG. Very limited success has been found in prior studies with UCBED cohorts^11,12^. Moreover, these studies either did not investigate populations of children or simply applied commercially available control systems that were built on research and refinement in populations with acquired amputations. Our work shows distinguishable and consistent patterns of muscle excitation in children with UCBED when they attempt to perform hand movements. We suggest that these findings warrant further investigation into techniques to best tune, adapt, and leverage existing advanced control techniques, such that they may be most effective in meeting the unique demand of children, most of whom will have a congenital upper limb absence^2^.

### Supplementary Information

Supplementary Figures.

## Data Availability

The data that support the findings of this study are available from Shriners Children's—Northern California but restrictions apply to the availability of these data, which were used under license for the current study, and so are not publicly available. Data are however available from the corresponding author upon reasonable request and with permission of Shriners Children’s.
