# Supplementary material for: Understanding the capacity of children with congenital unilateral below-elbow deficiency to actuate their affected muscles
Source: Sci Rep. 2024 Feb 24;14:4563. doi: 10.1038/s41598-024-54952-7 (PMC10894282; doi:10.1038/s41598-024-54952-7)
Supplement: Supplementary file 1 — Supplementary Figures. [file 41598_2024_54952_MOESM1_ESM.pdf]

Understanding the Capacity of Children with Congenital Unilateral Below-Elbow Deficiency to Actuate Their Affected Muscles

Marcus A Battraw[1], Justin Fitzgerald[2,3,4], Michelle A James[5,6], Anita M Bagley[5,6], Wilsaan M Joiner[3,7], Jonathon S Schofield*[1]

[1]Department of Mechanical and Aerospace Engineering, University of California, Davis, Davis CA, USA

[2]Department of Biomedical Engineering, University of California, Davis, Davis CA, USA

[3]Department of Neurobiology, Physiology and Behavior, University of California, Davis, Davis CA, USA

[4]Clinical and Translational Science Center, University of California, Davis Health; Sacramento, CA, USA

[5]Shriners Children's – Northern California, Sacramento CA, USA

[6]Department of Orthopaedic Surgery, University of California Davis Health, Sacramento CA, USA

[7]Department of Neurology, University of California Davis Health, Sacramento CA, USA

*** Correspondence:**
Jonathon Schofield
jschofield@ucdavis.edu

RMS and MNF muscle excitation

Participant: SHR-A

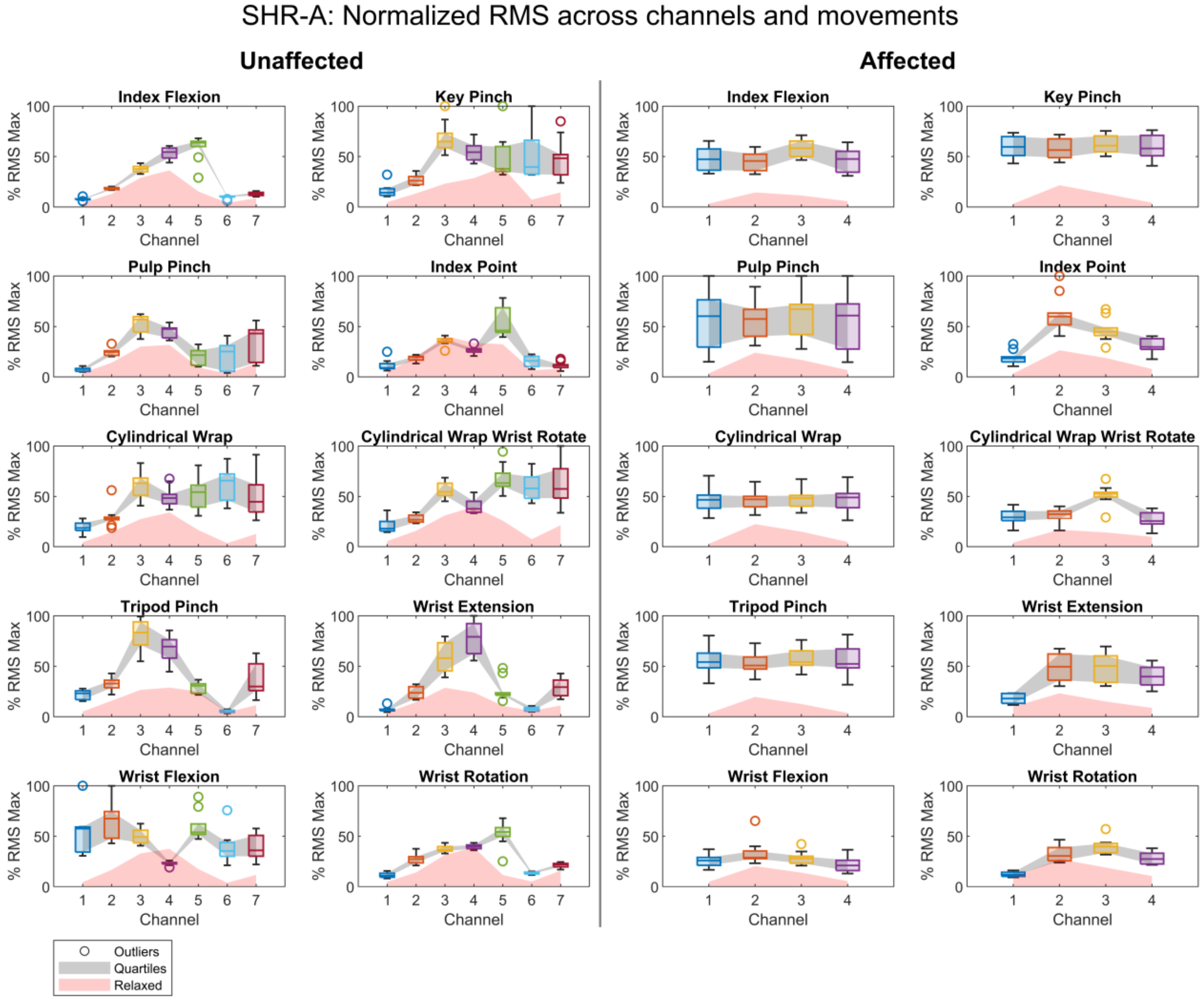

Supplementary Figure S1. The box and whisker plots provide a visualization of the RMS muscle excitation patterns and relaxed states seen for the various hand movements across the unaffected and affected limbs for participant SHR-A.

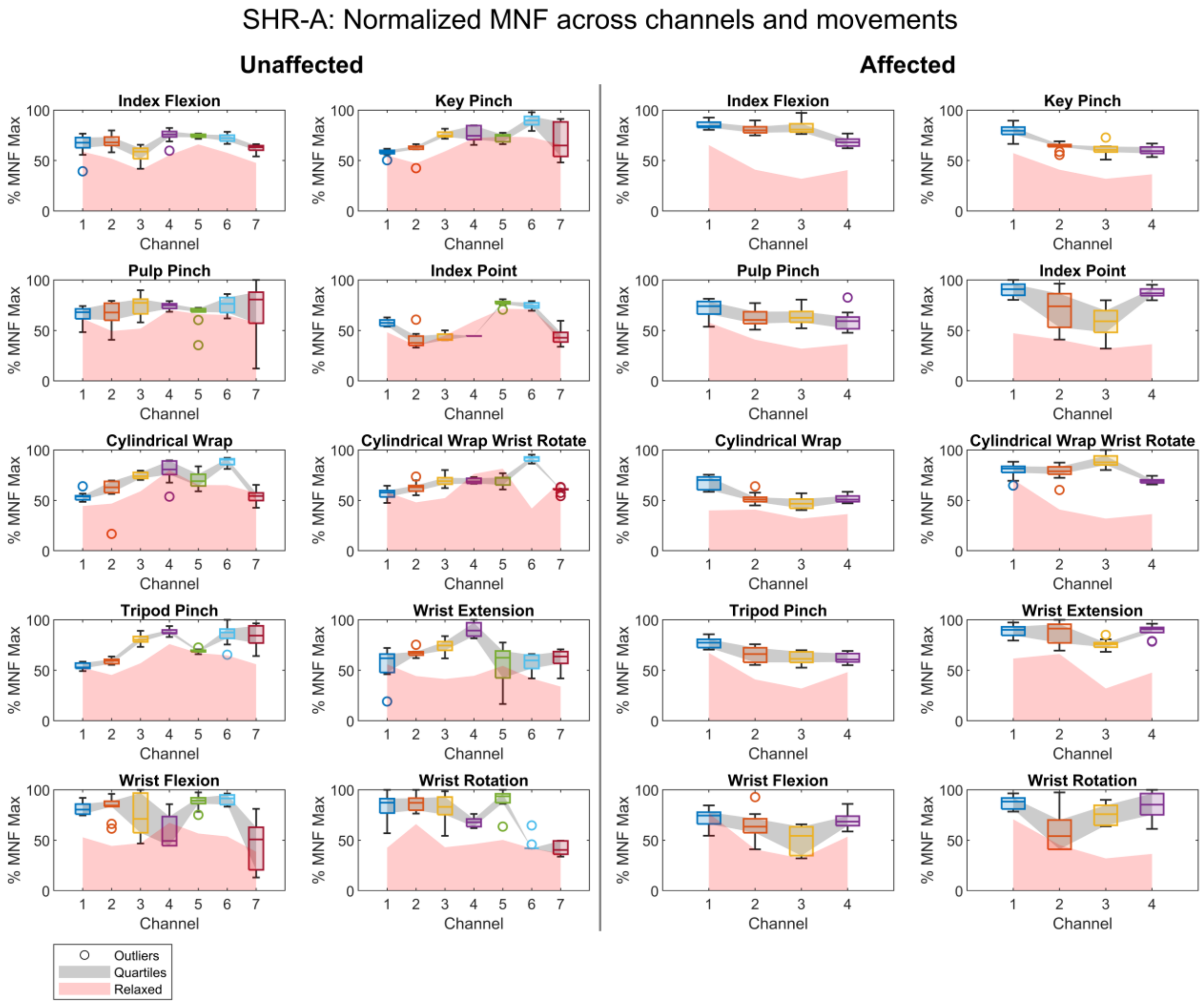

Supplementary Figure S2. The box and whisker plots provide a visualization of the MNF muscle excitation patterns and relaxed states seen for the various hand movements across the unaffected and affected limbs for participant SHR-A.

Participant: SHR-B

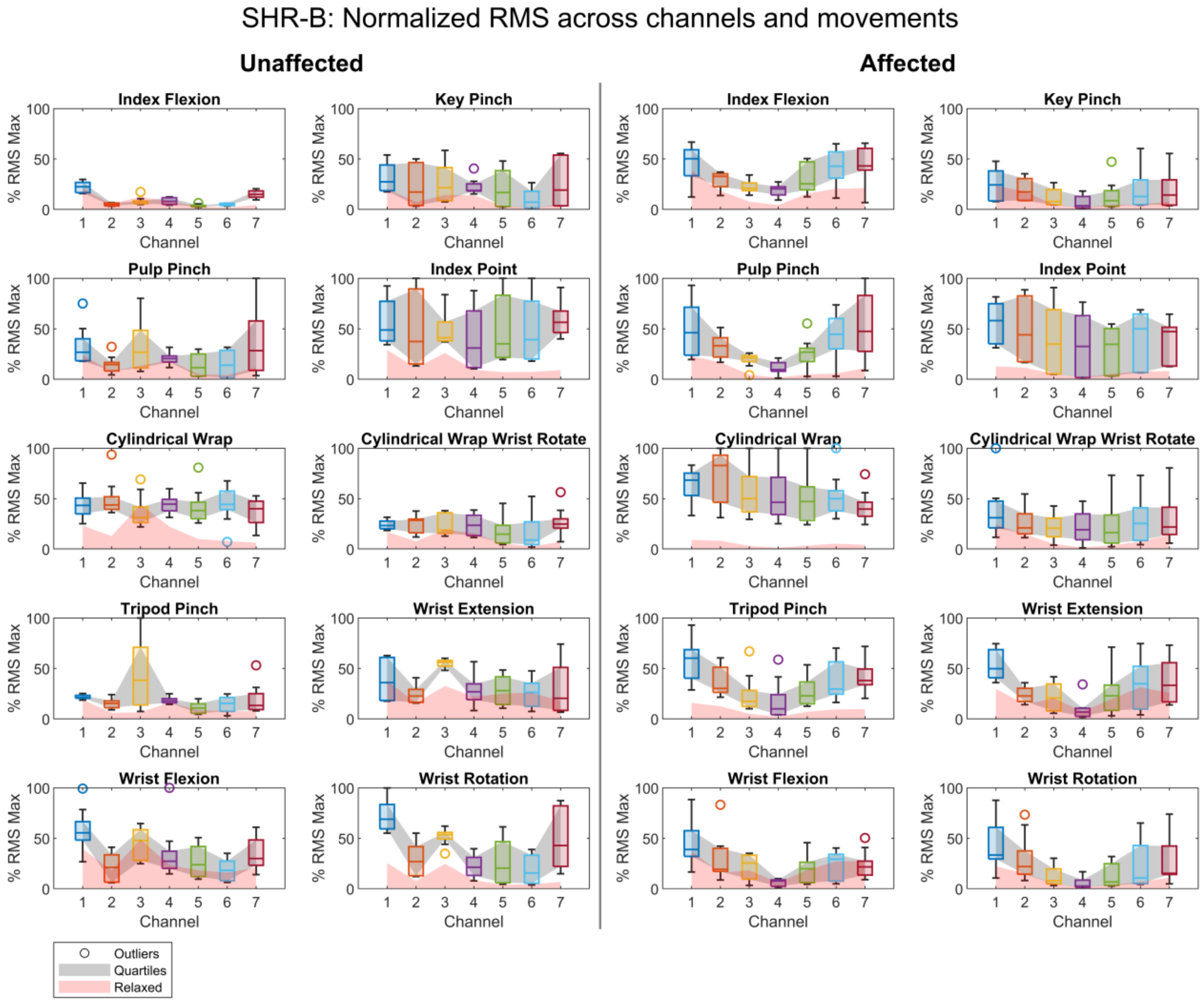

Supplementary Figure S3. The box and whisker plots provide a visualization of the RMS muscle excitation patterns and relaxed states seen for the various hand movements across the unaffected and affected limbs for participant SHR-B.

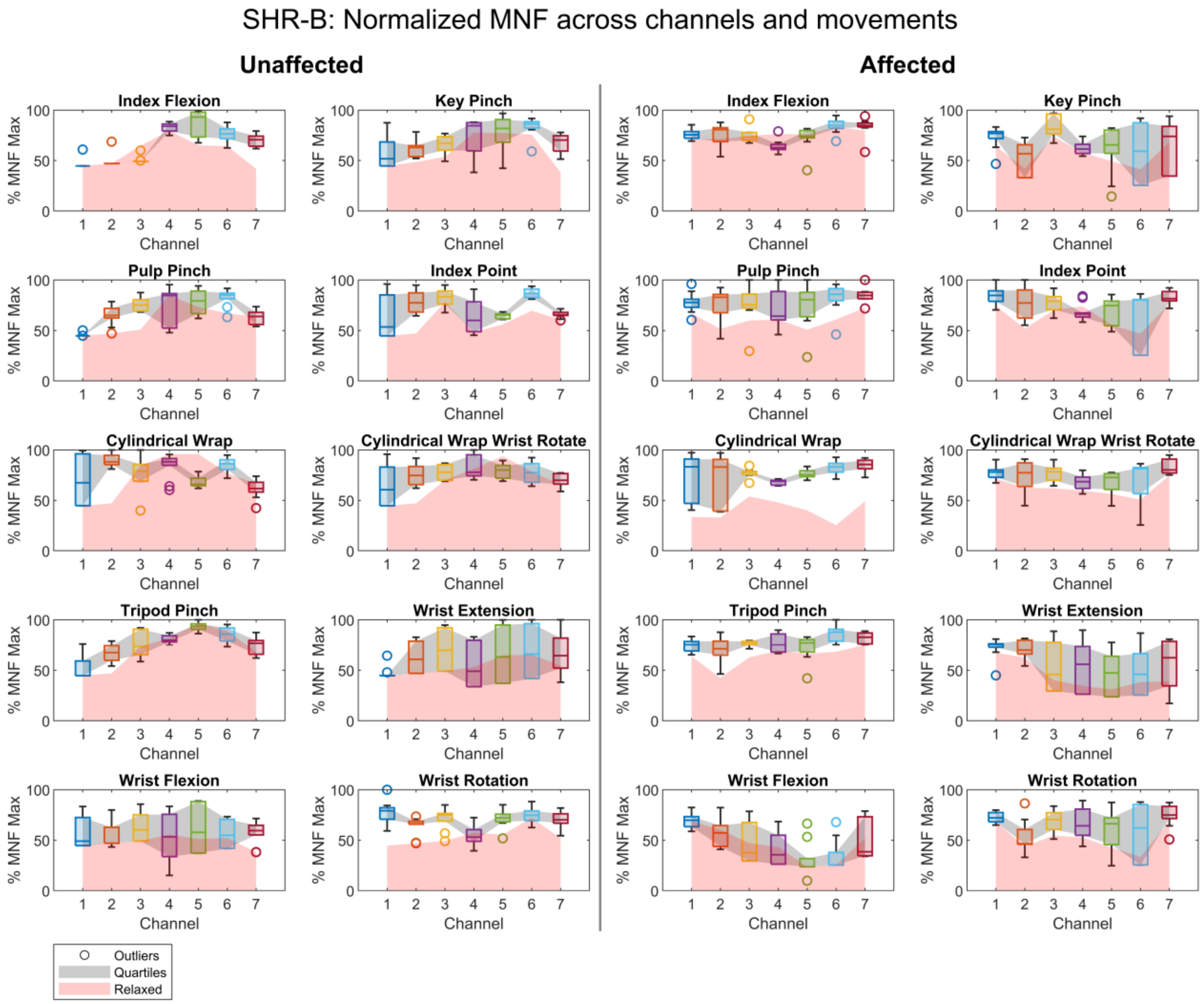

Supplementary Figure S4. The box and whisker plots provide a visualization of the MNF muscle excitation patterns and relaxed states seen for the various hand movements across the unaffected and affected limbs for participant SHR-B.

Participant: SHR-C

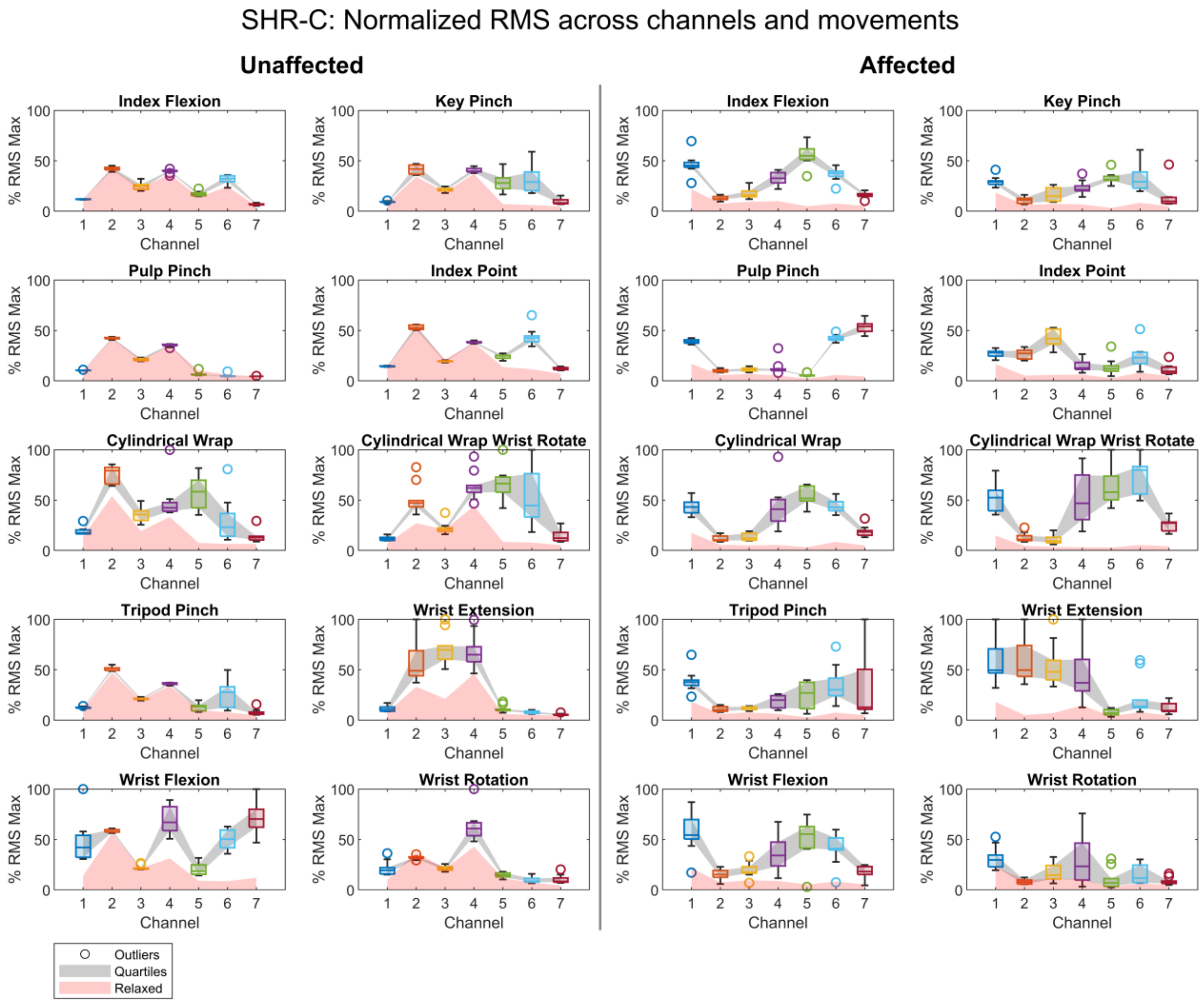

Supplementary Figure S5. The box and whisker plots provide a visualization of the RMS muscle excitation patterns and relaxed states seen for the various hand movements across the unaffected and affected limbs for participant SHR-C.

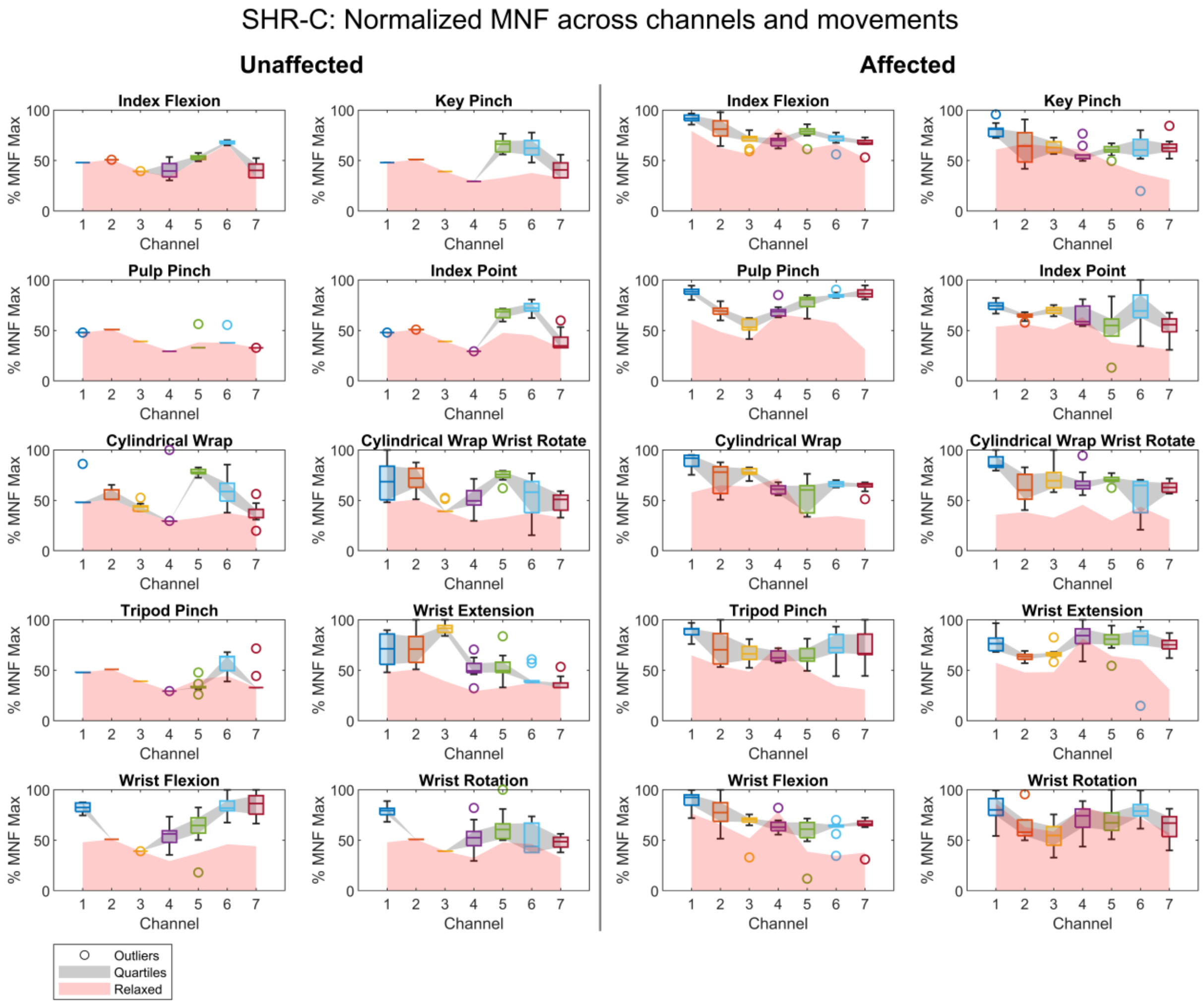

Supplementary Figure S6. The box and whisker plots provide a visualization of the MNF muscle excitation patterns and relaxed states seen for the various hand movements across the unaffected and affected limbs for participant SHR-C.

Participant: SHR-D

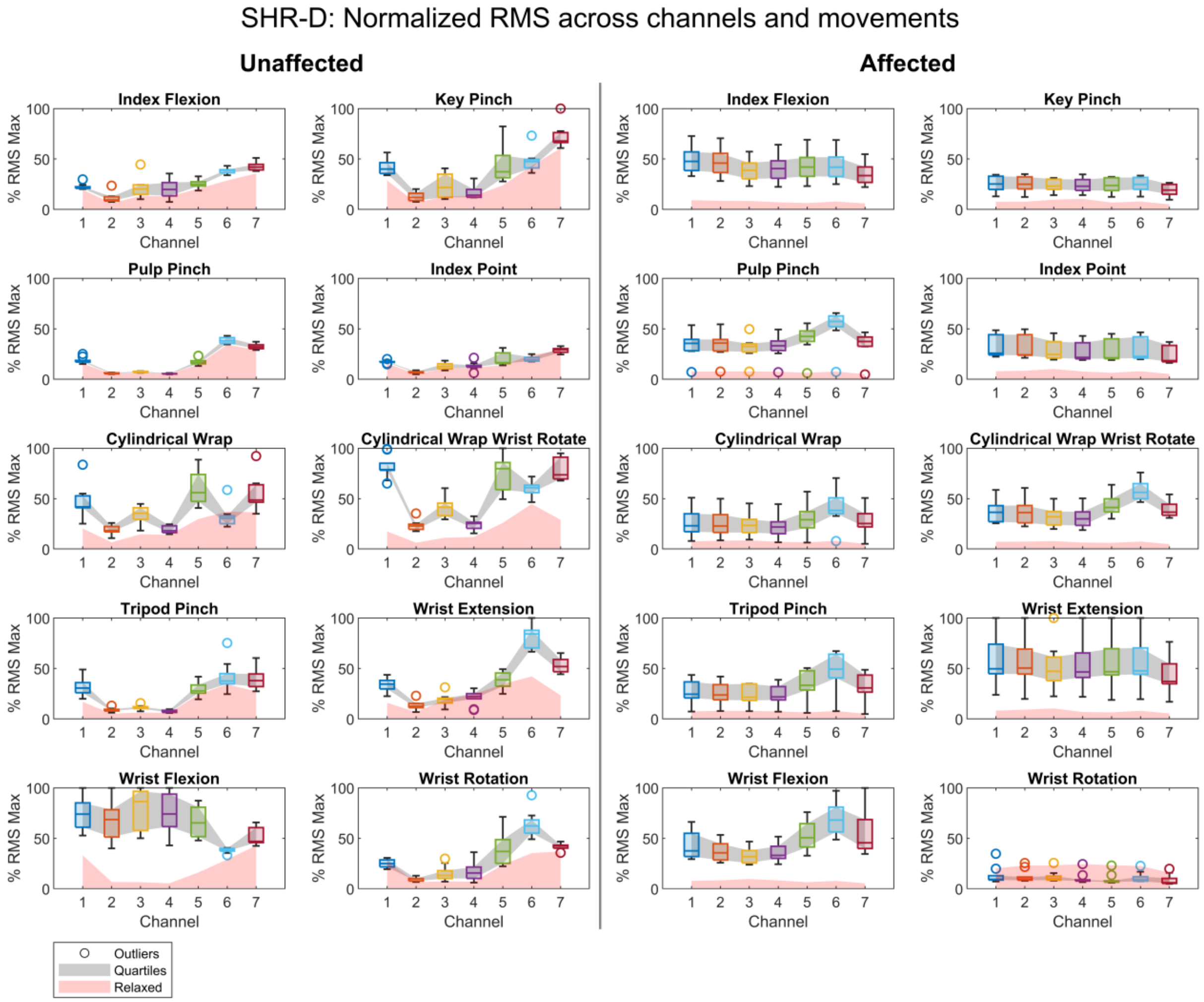

Supplementary Figure S7. The box and whisker plots provide a visualization of the RMS muscle excitation patterns and relaxed states seen for the various hand movements across the unaffected and affected limbs for participant SHR-D.

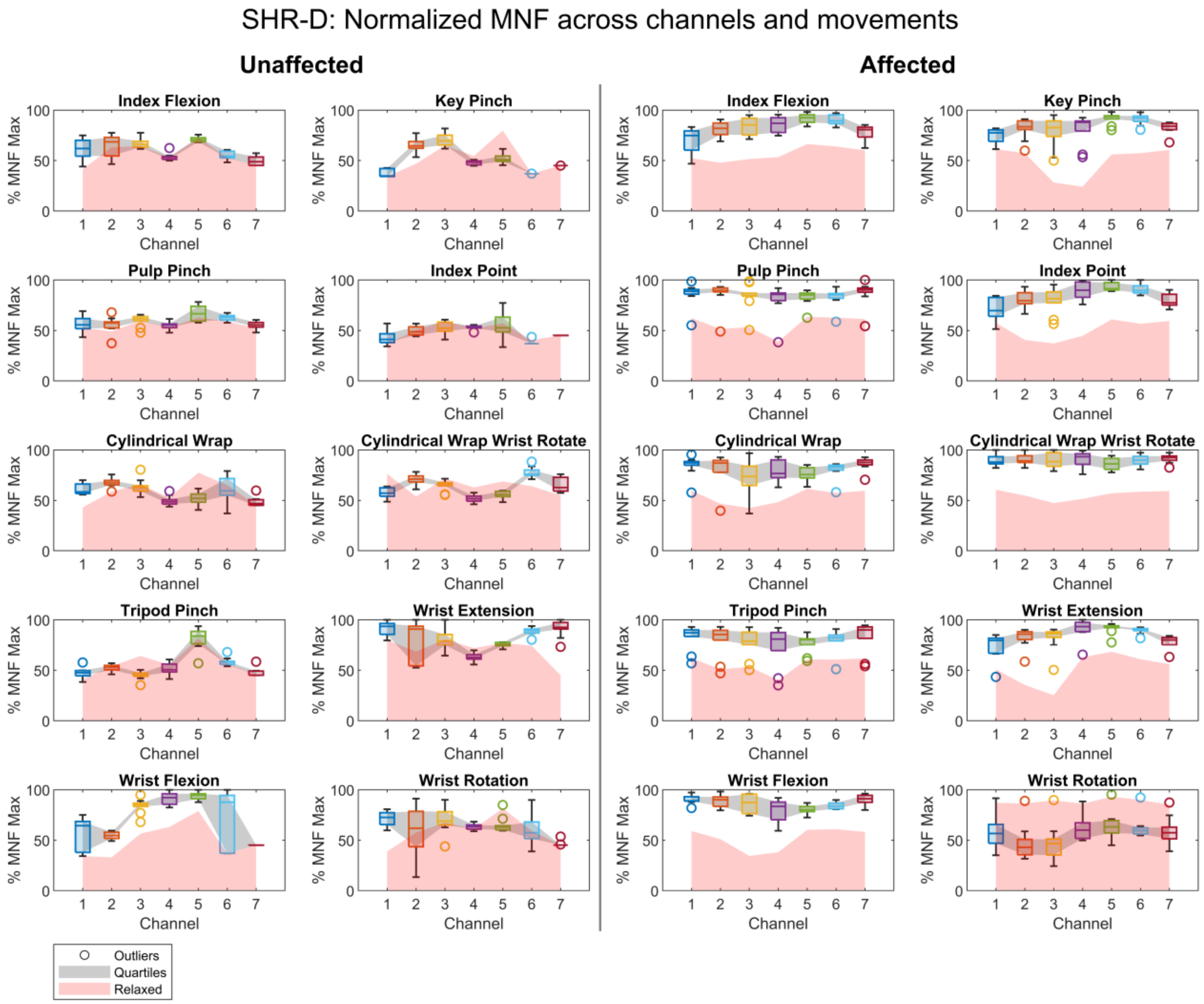

Supplementary Figure S8. The box and whisker plots provide a visualization of the MNF muscle excitation patterns and relaxed states seen for the various hand movements across the unaffected and affected limbs for participant SHR-D.

Participant: SHR-E

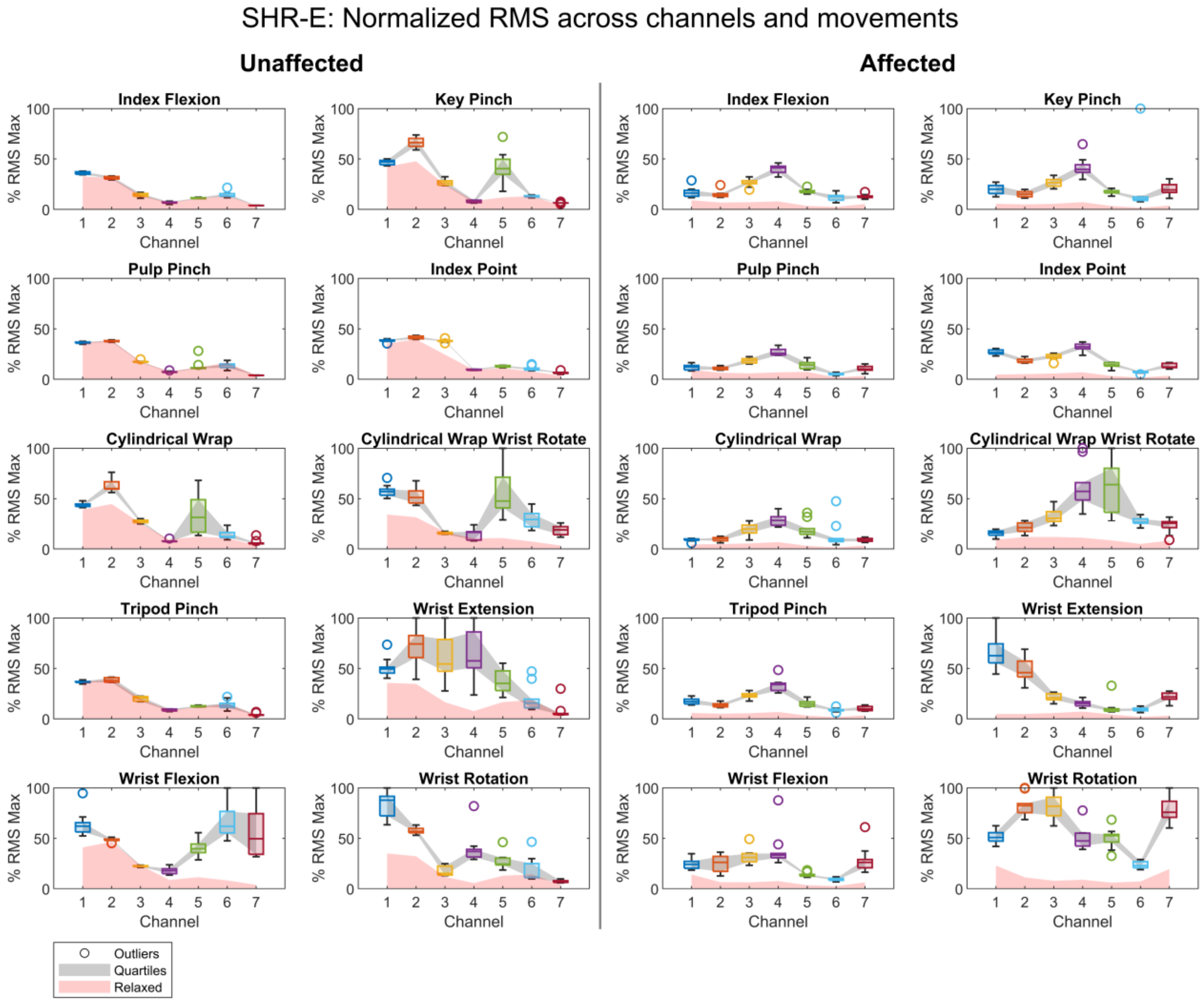

Supplementary Figure S9. The box and whisker plots provide a visualization of the RMS muscle excitation patterns and relaxed states seen for the various hand movements across the unaffected and affected limbs for participant SHR-E.

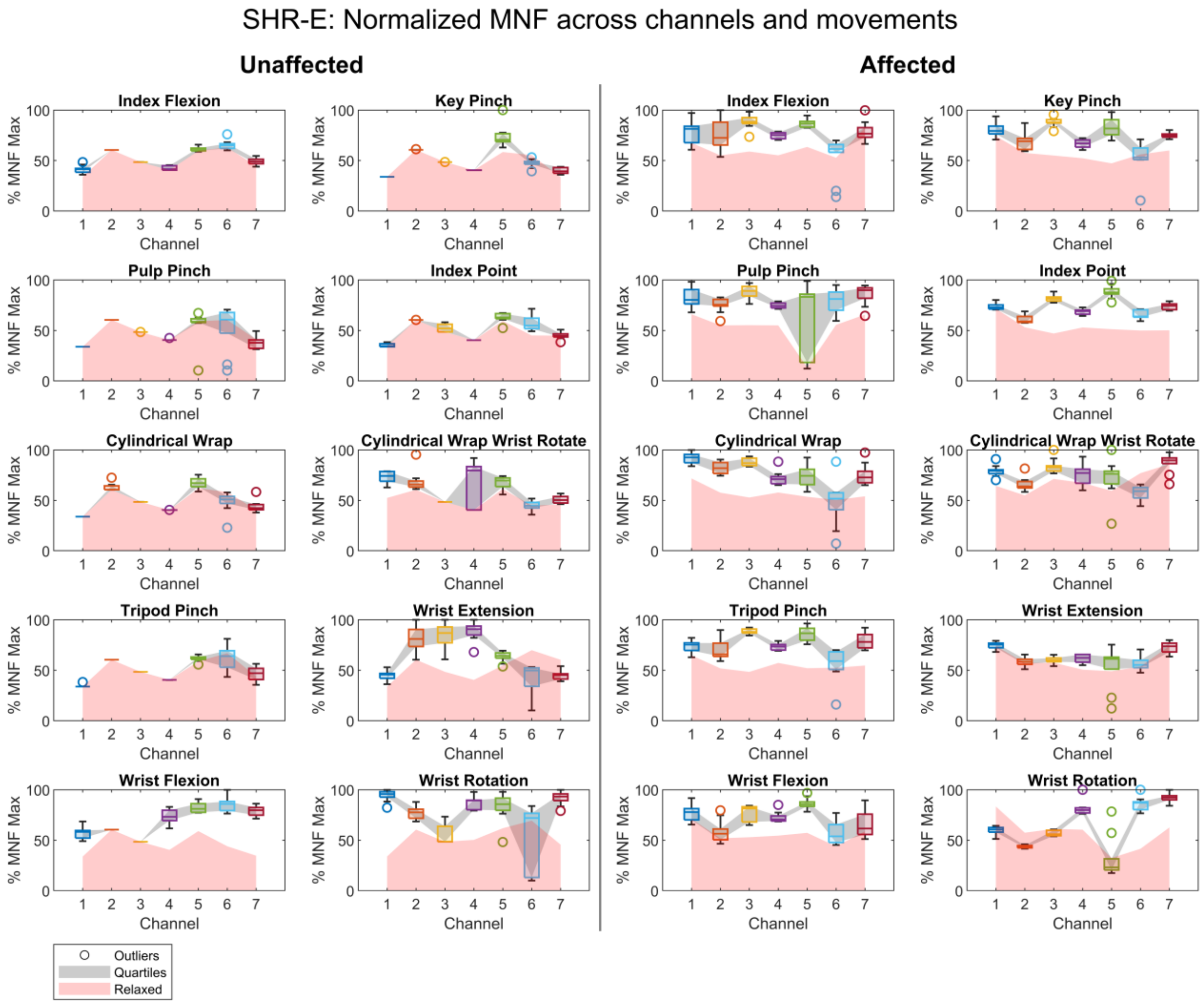

Supplementary Figure S10. The box and whisker plots provide a visualization of the MNF muscle excitation patterns and relaxed states seen for the various hand movements across the unaffected and affected limbs for participant SHR-E.

Participant: SHR-F

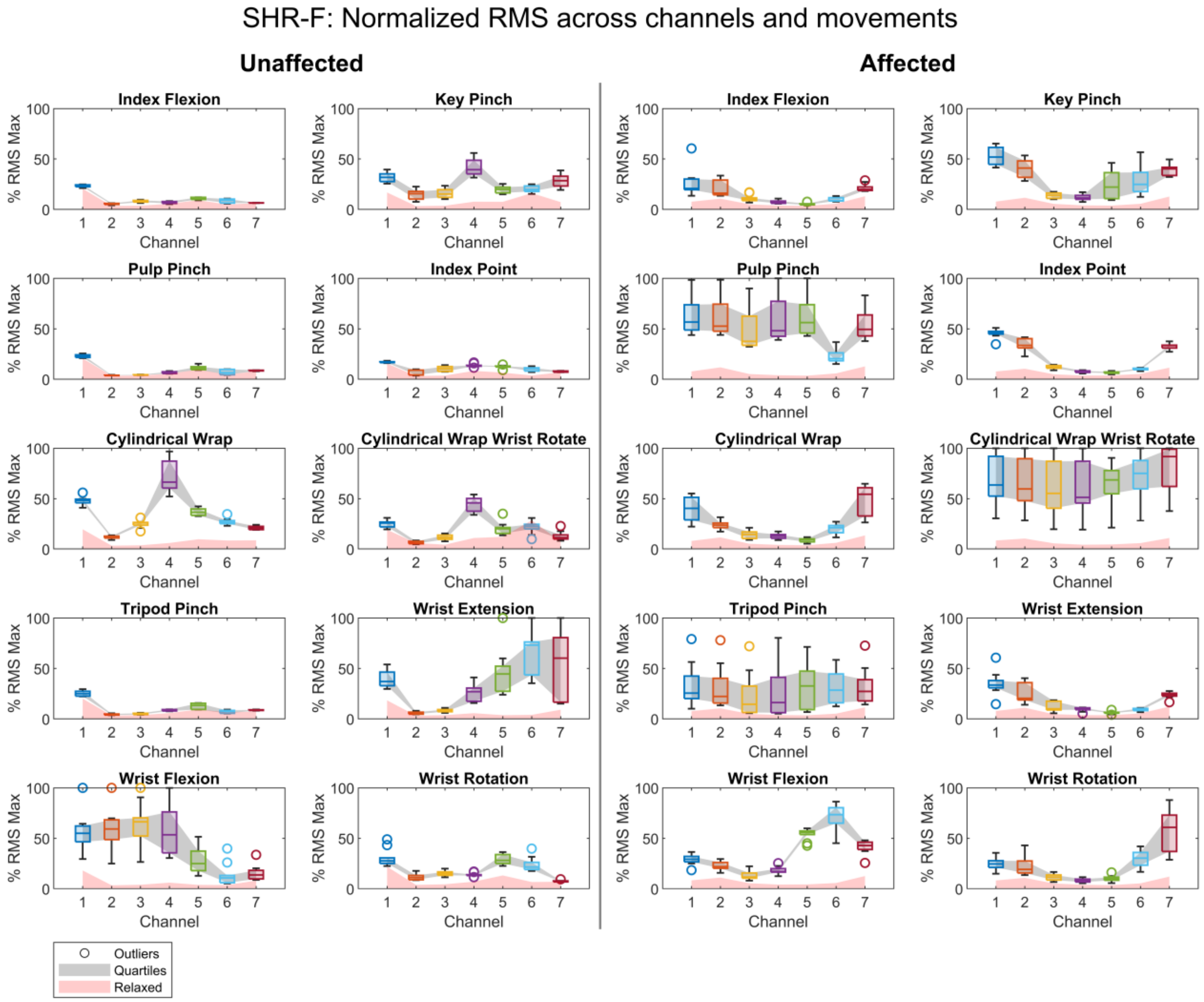

Supplementary Figure S11. The box and whisker plots provide a visualization of the RMS muscle excitation patterns and relaxed states seen for the various hand movements across the unaffected and affected limbs for participant SHR-F.

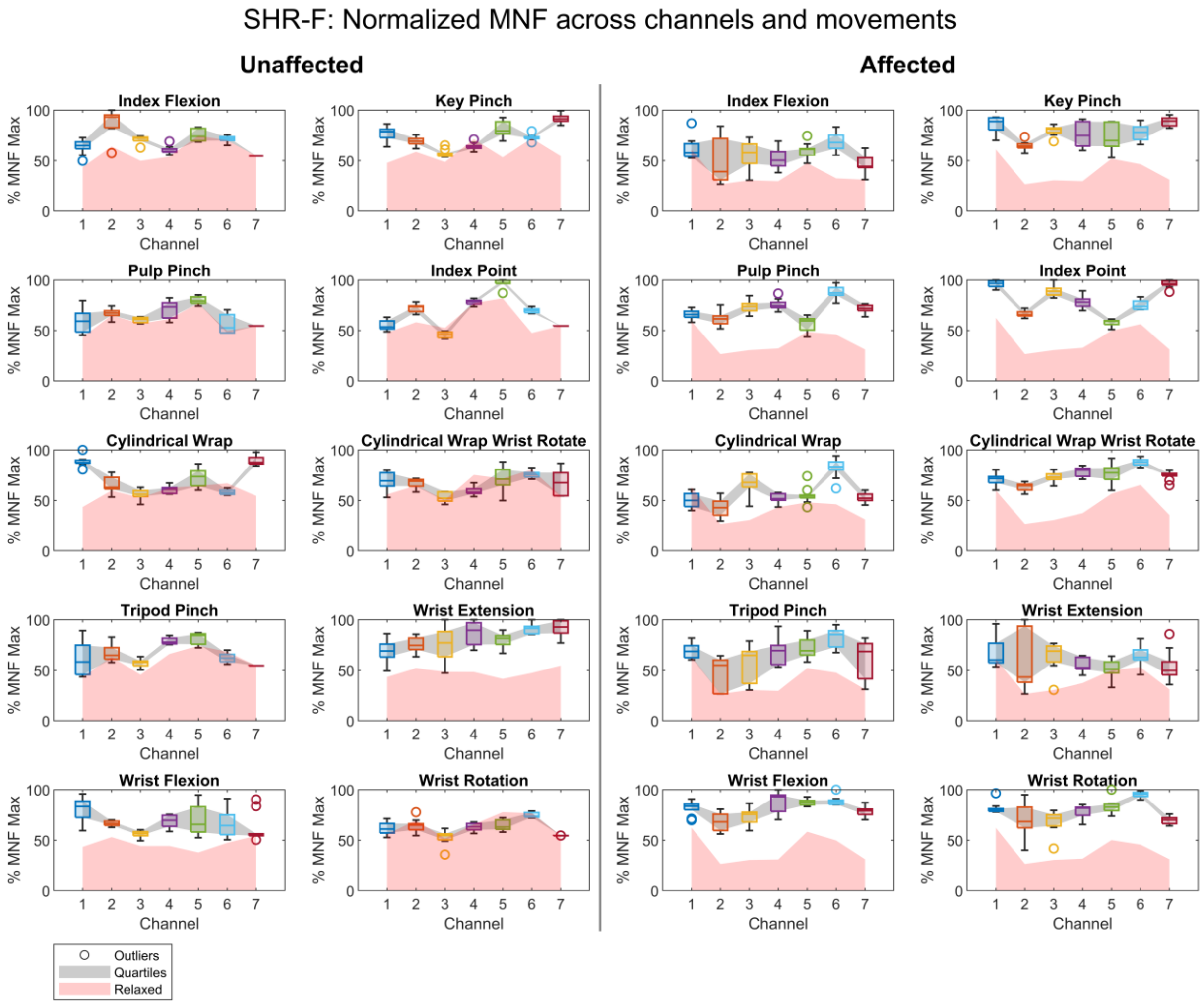

Supplementary Figure S12. The box and whisker plots provide a visualization of the MNF muscle excitation patterns and relaxed states seen for the various hand movements across the unaffected and affected limbs for participant SHR-F.

Participant: SHR-G

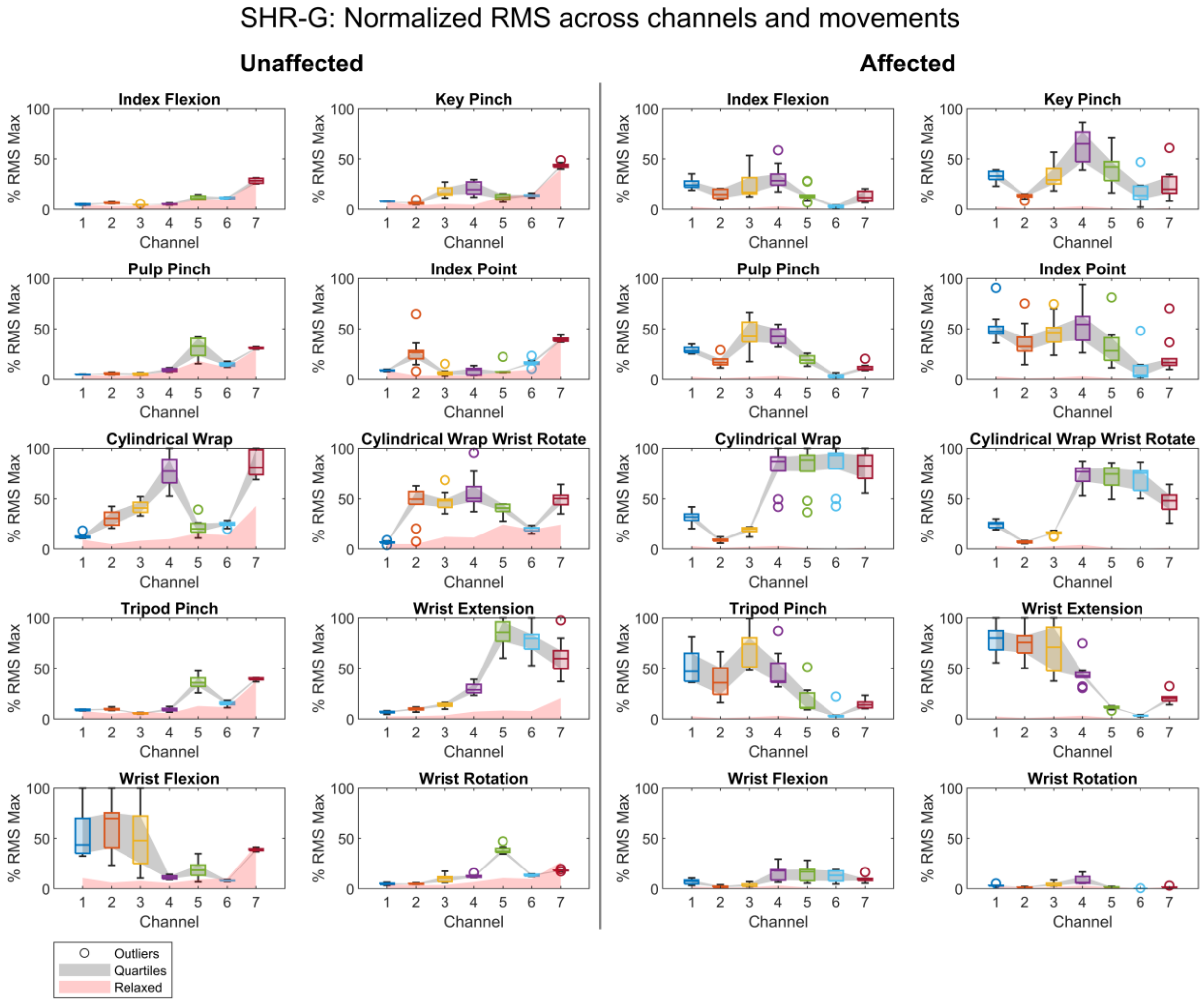

Supplementary Figure S13. The box and whisker plots provide a visualization of the RMS muscle excitation patterns and relaxed states seen for the various hand movements across the unaffected and affected limbs for participant SHR-G.

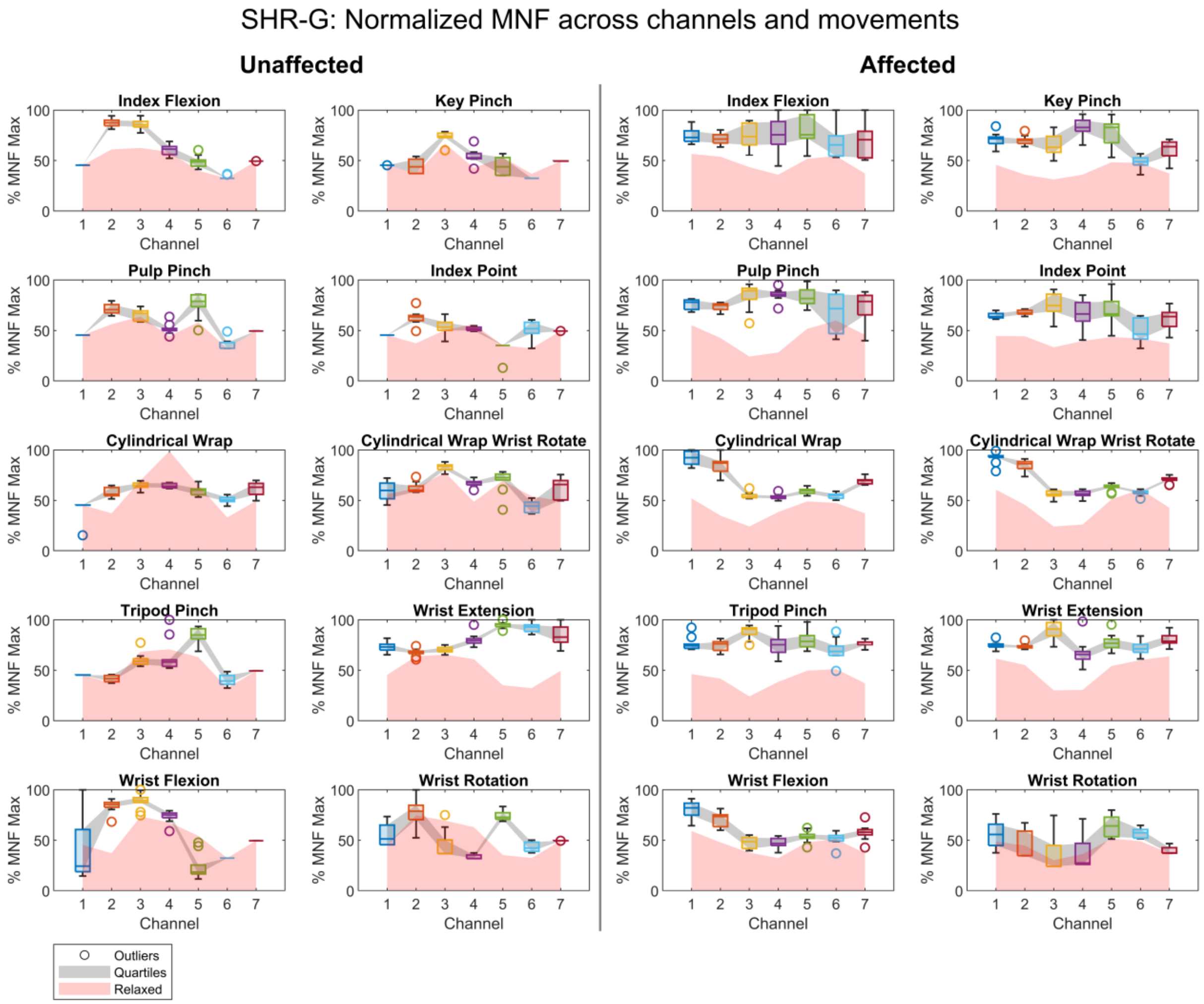

Supplementary Figure S14. The box and whisker plots provide a visualization of the MNF muscle excitation patterns and relaxed states seen for the various hand movements across the unaffected and affected limbs for participant SHR-G.

Participant: SHR-H

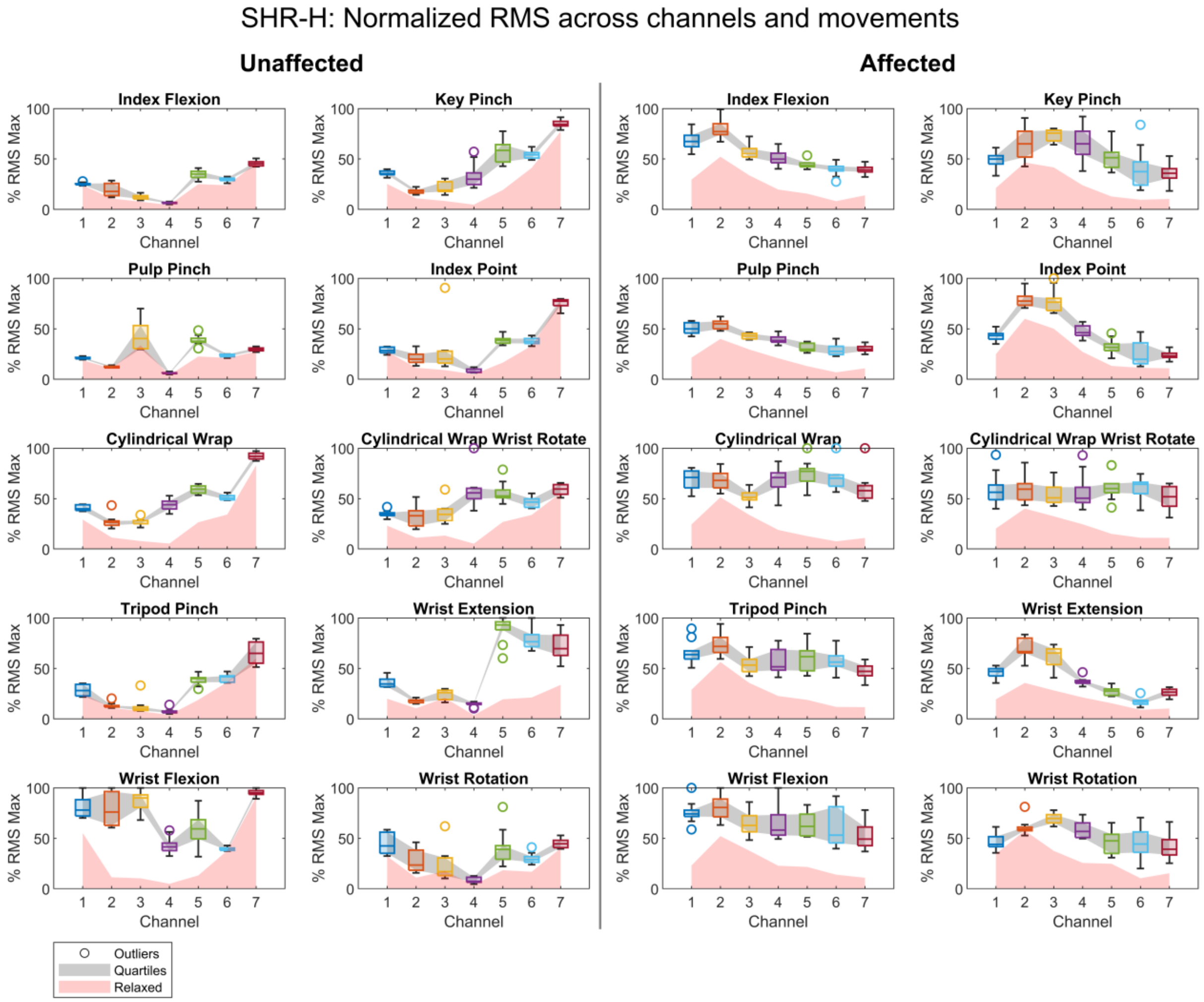

Supplementary Figure S15. The box and whisker plots provide a visualization of the RMS muscle excitation patterns and relaxed states seen for the various hand movements across the unaffected and affected limbs for participant SHR-H.

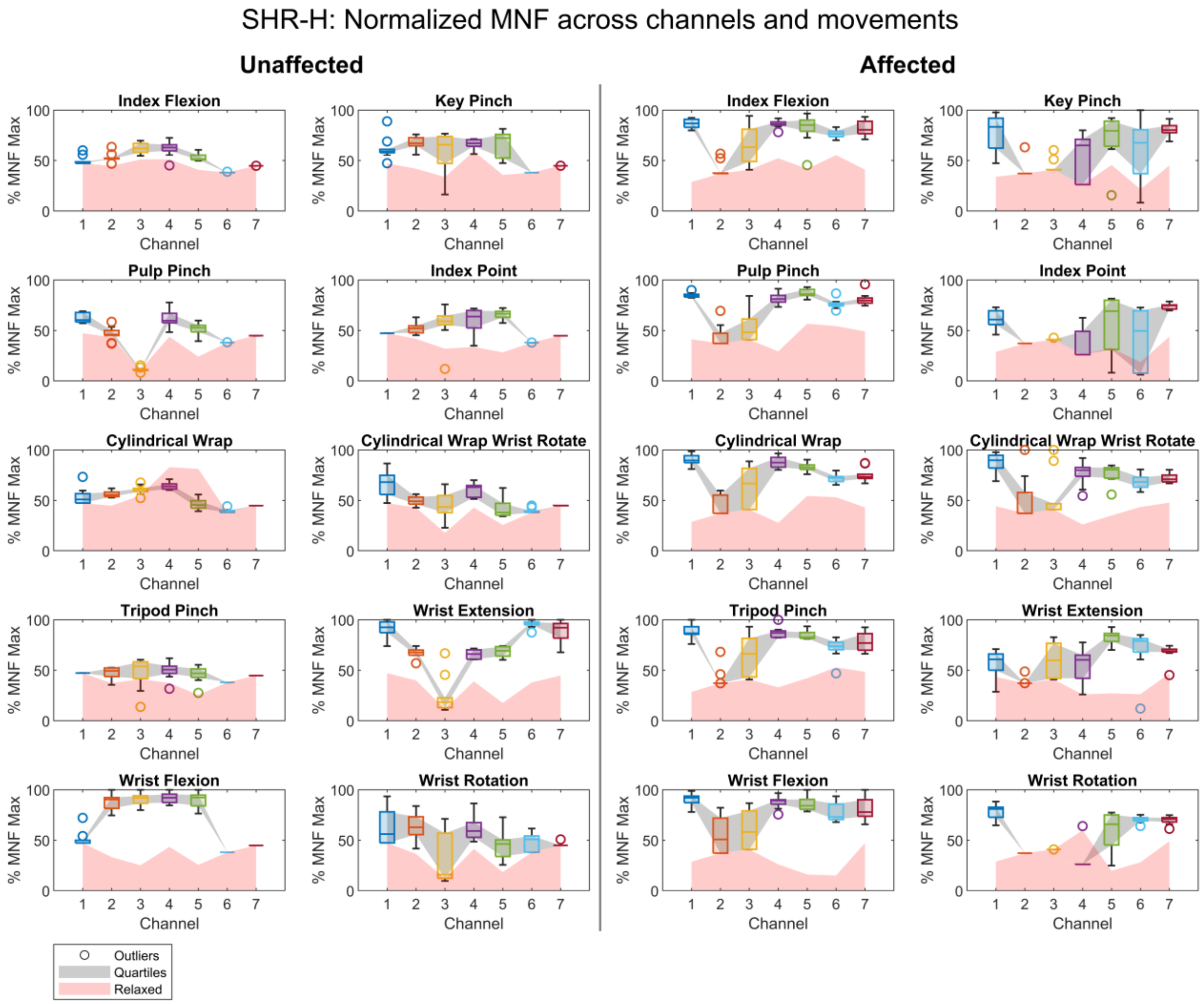

Supplementary Figure S16. The box and whisker plots provide a visualization of the MNF muscle excitation patterns and relaxed states seen for the various hand movements across the unaffected and affected limbs for participant SHR-H.

Participant: SHR-I

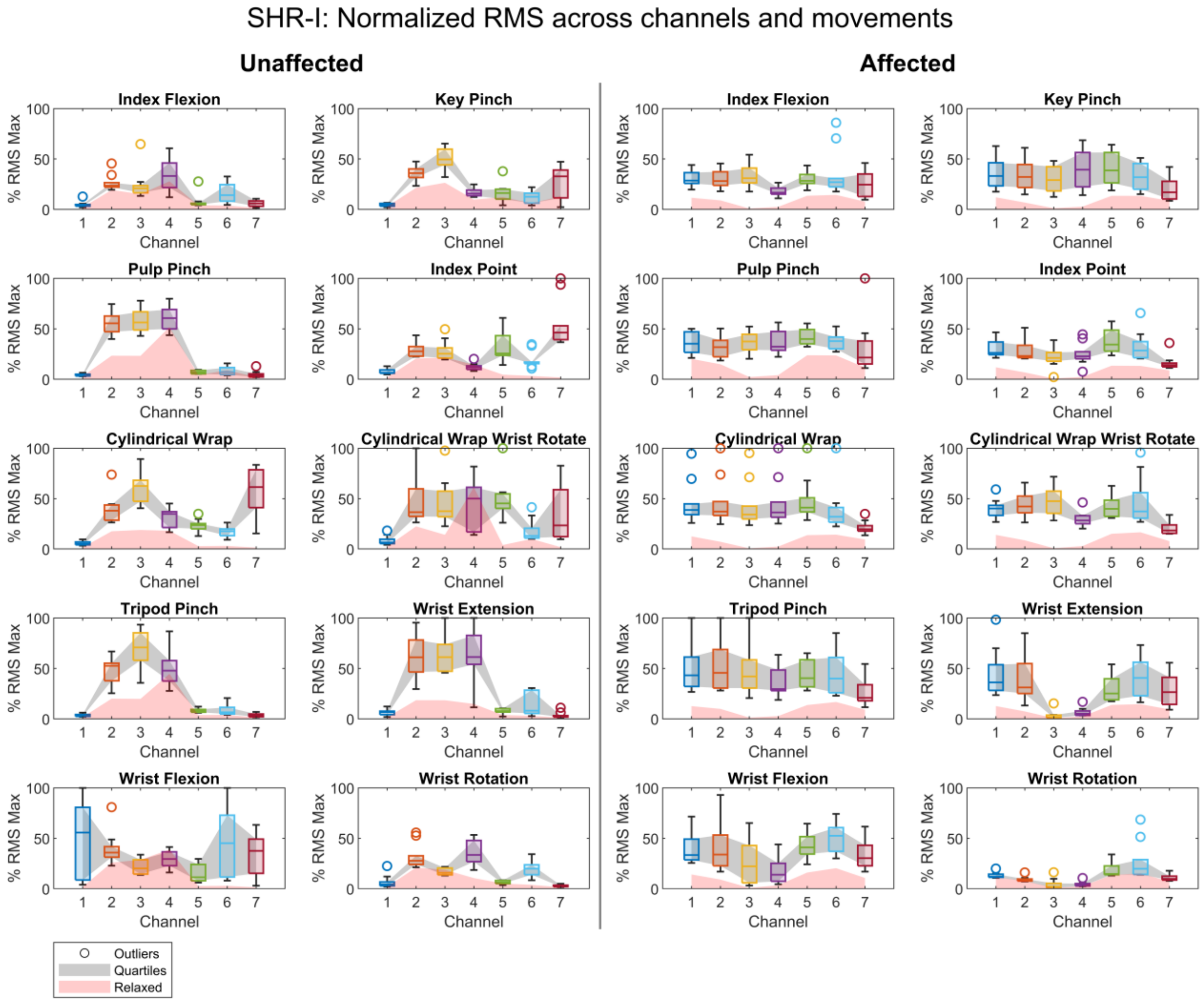

Supplementary Figure S17. The box and whisker plots provide a visualization of the RMS muscle excitation patterns and relaxed states seen for the various hand movements across the unaffected and affected limbs for participant SHR-I.

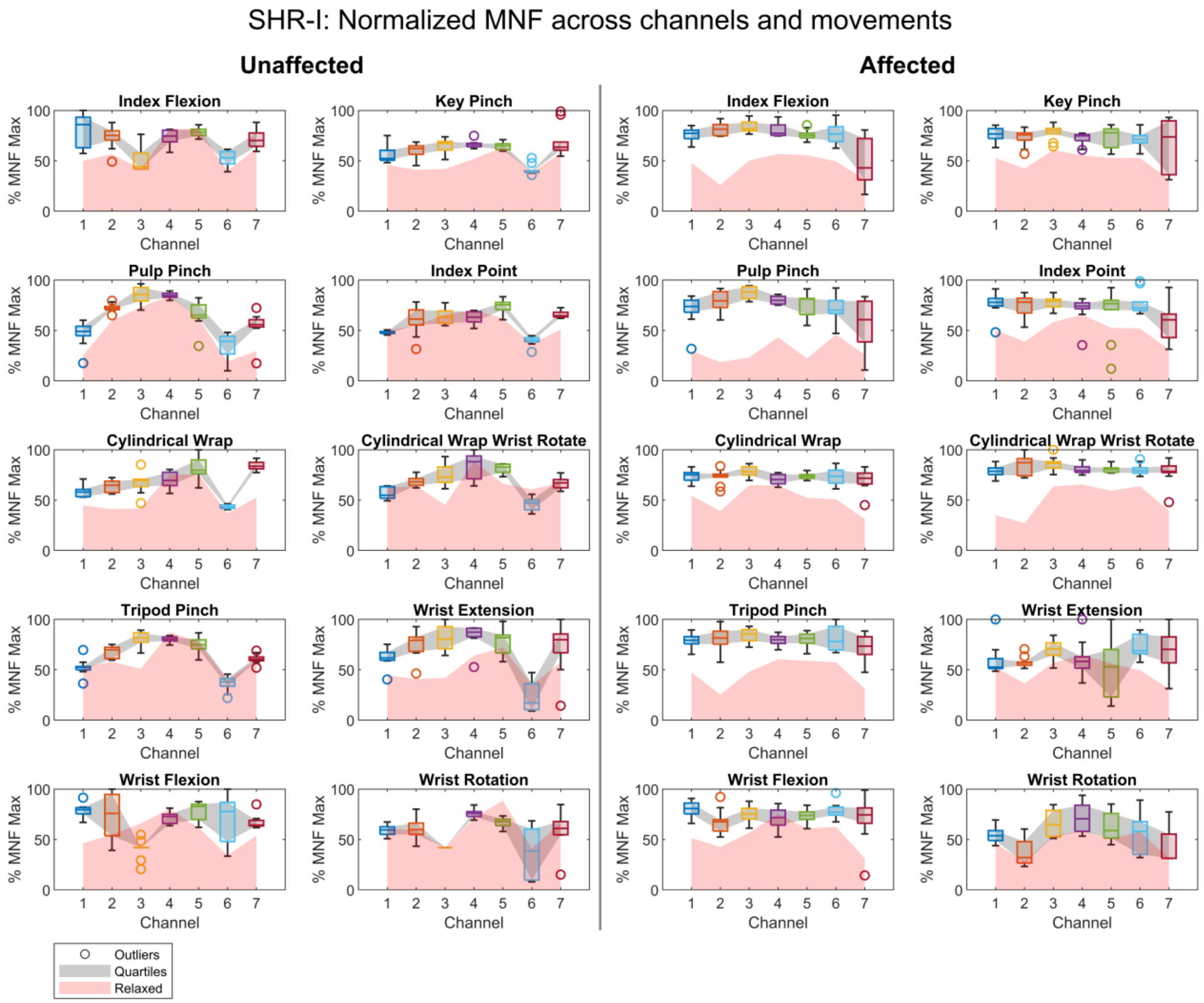

Supplementary Figure S18. The box and whisker plots provide a visualization of the MNF muscle excitation patterns and relaxed states seen for the various hand movements across the unaffected and affected limbs for participant SHR-I.

Multidimensional scaling and representational dissimilarity matrices

Participant: SHR-A

(a)

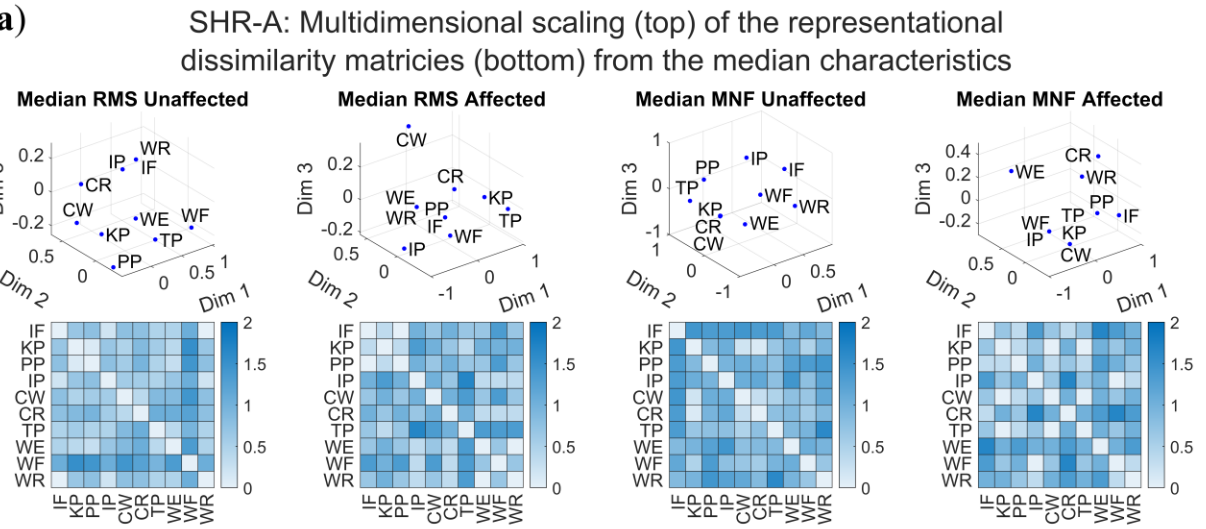

(b)

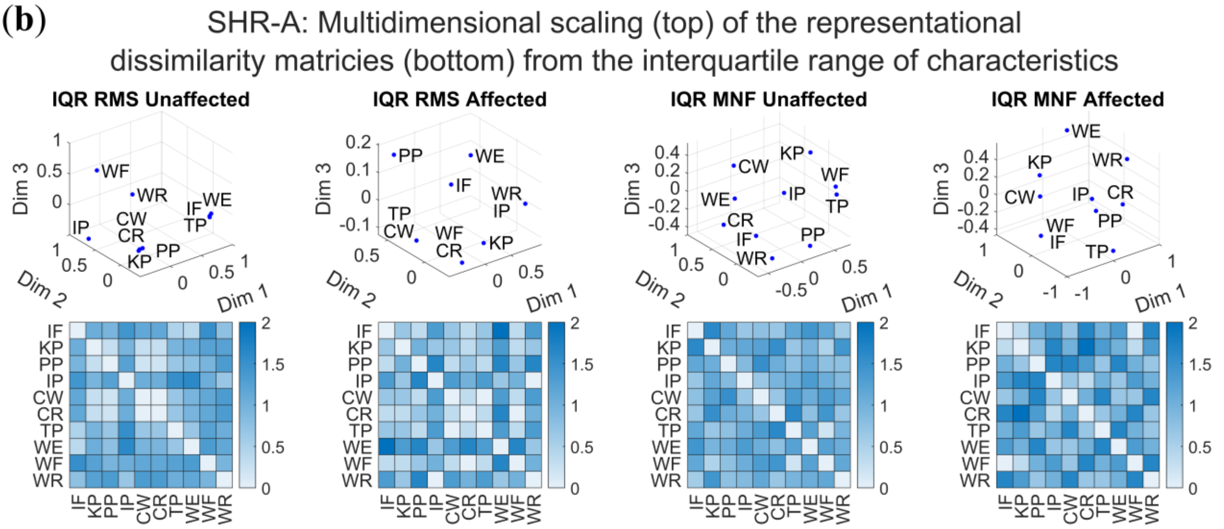

Supplementary Figure S19. Visual representation of the correlation distances between hand movements for the amplitude and spread of measurement characteristics of SHR-A. The multidimensional scaling plots are provided in three dimensions corresponding to the representational dissimilarity matrices for (**a**) the median RMS and MNF characteristics and (**b**) the RMS and MNF interquartile range (IQR).

Participant: SHR-B

(**a**)

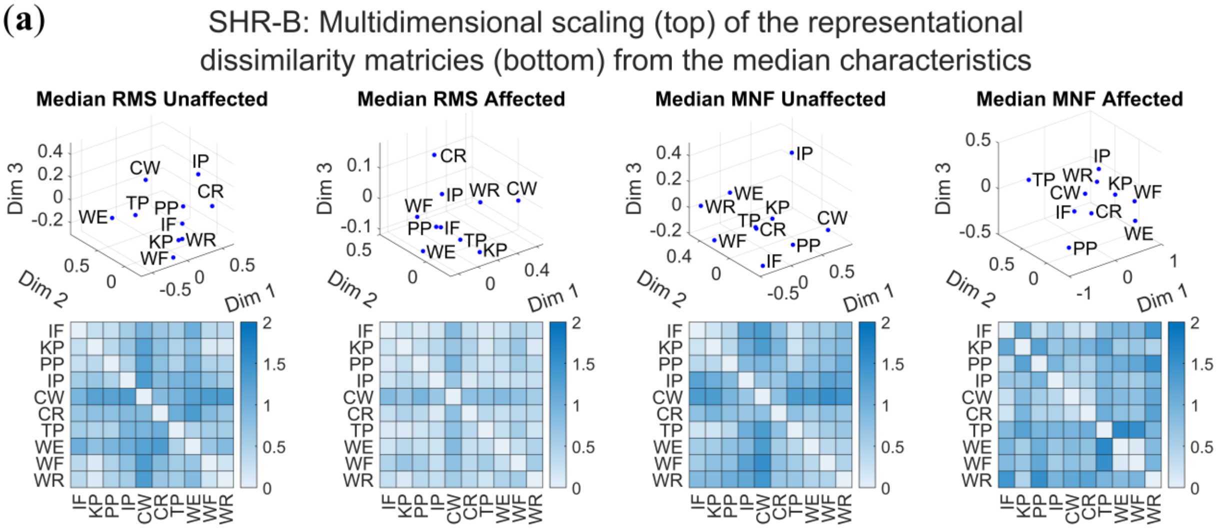

(**b**)

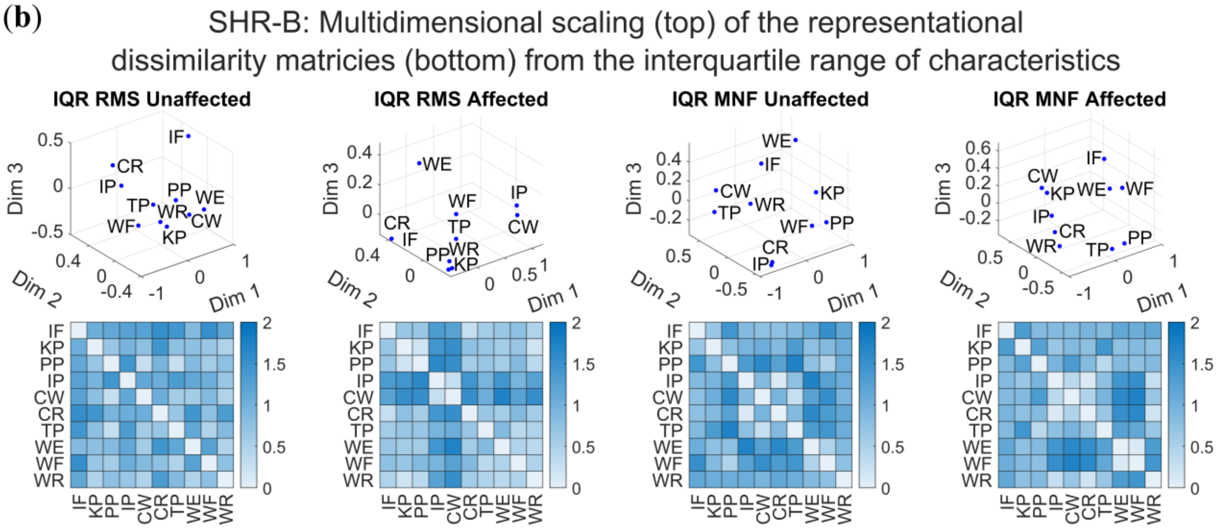

Supplementary Figure S20. Visual representation of the correlation distances between hand movements for the amplitude and spread of measurement characteristics of SHR-B. The multidimensional scaling plots are provided in three dimensions corresponding to the representational dissimilarity matrices for (**a**) the median RMS and MNF characteristics and (**b**) the RMS and MNF interquartile range (IQR).

Participant: SHR-C

(**a**)

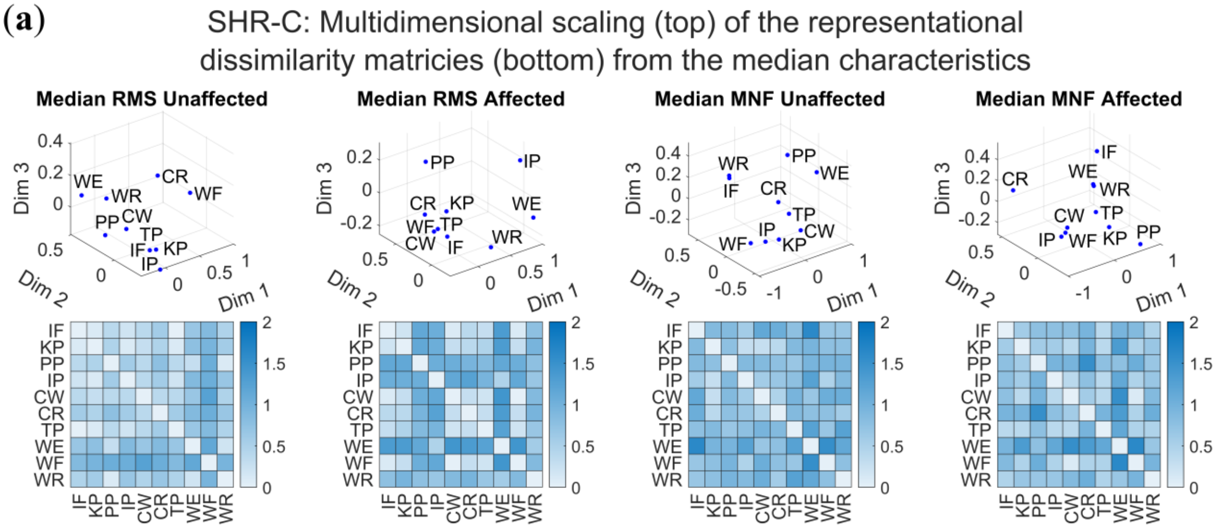

(**b**)

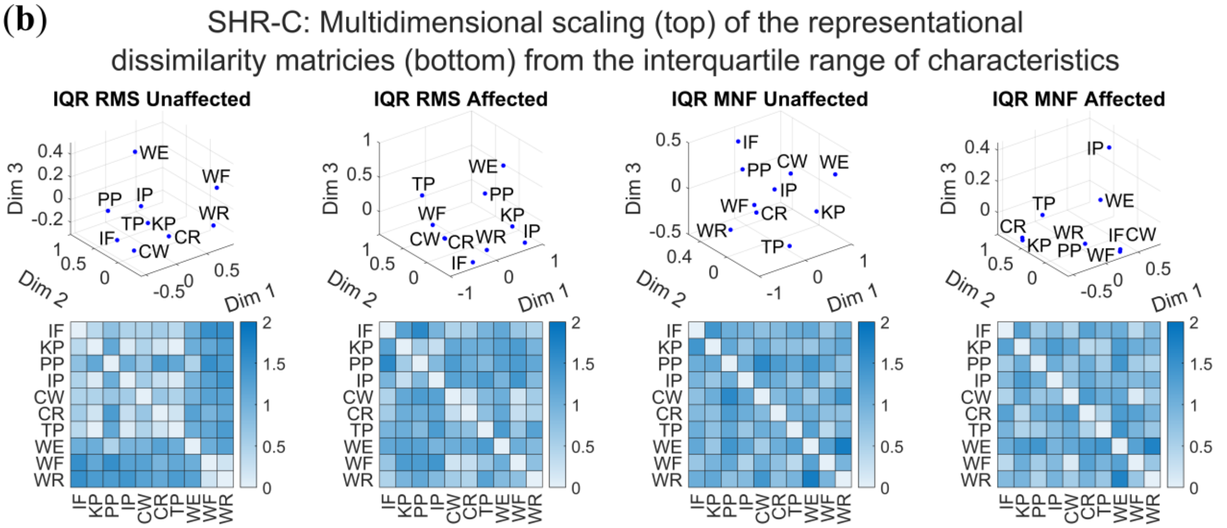

Supplementary Figure S21. Visual representation of the correlation distances between hand movements for the amplitude and spread of measurement characteristics of SHR-C. The multidimensional scaling plots are provided in three dimensions corresponding to the representational dissimilarity matrices for (**a**) the median RMS and MNF characteristics and (**b**) the RMS and MNF interquartile range (IQR).

Participant: SHR-D

(a)

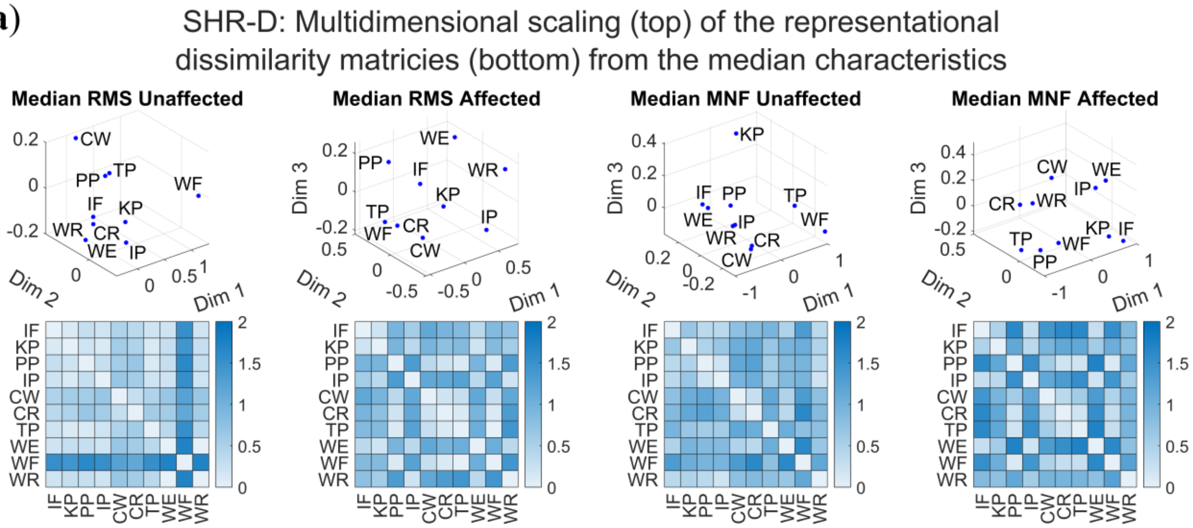

(b)

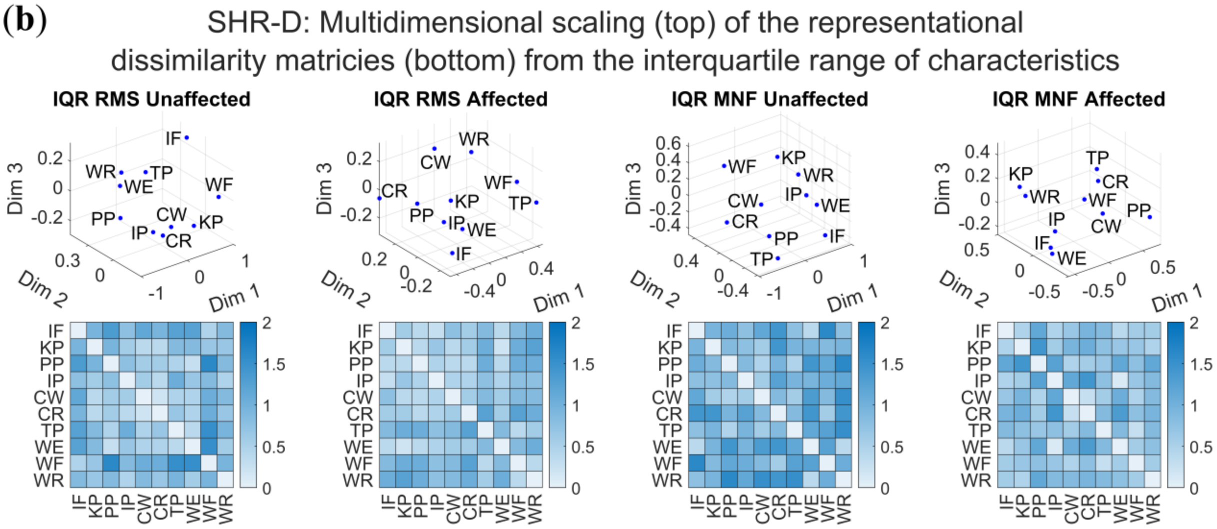

Supplementary Figure S22. Visual representation of the correlation distances between hand movements for the amplitude and spread of measurement characteristics of SHR-D. The multidimensional scaling plots are provided in three dimensions corresponding to the representational dissimilarity matrices for (**a**) the median RMS and MNF characteristics and (**b**) the RMS and MNF interquartile range (IQR).

Participant: SHR-E

(**a**)

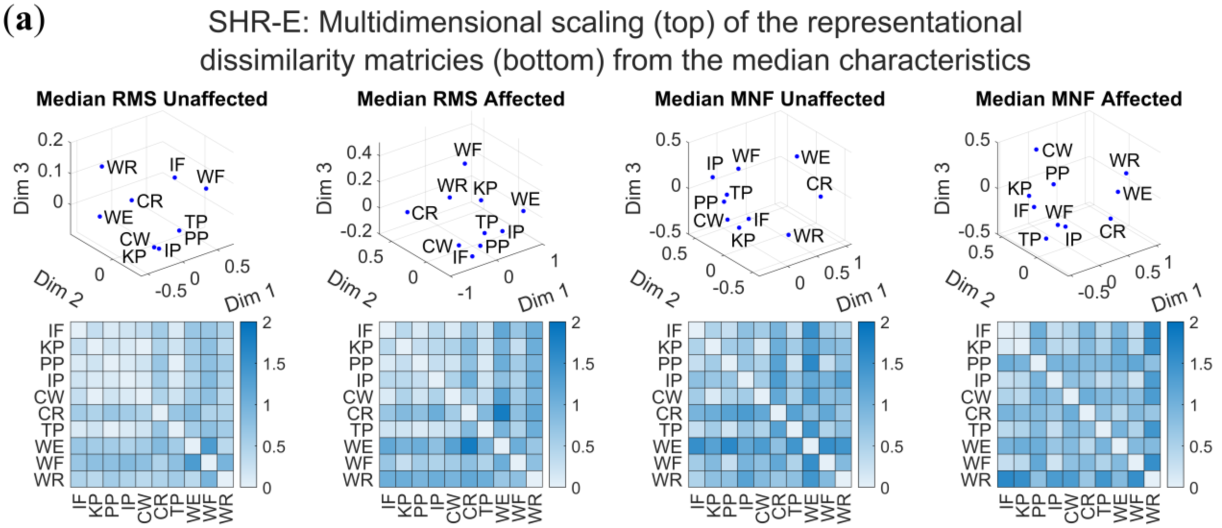

(**b**)

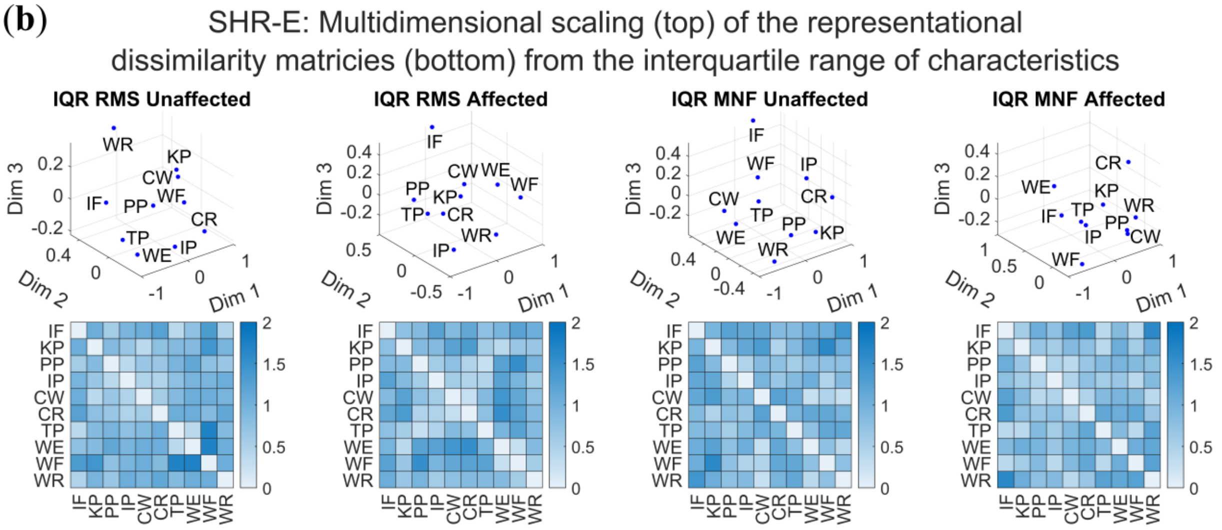

Supplementary Figure S23. Visual representation of the correlation distances between hand movements for the amplitude and spread of measurement characteristics of SHR-E. The multidimensional scaling plots are provided in three dimensions corresponding to the representational dissimilarity matrices for (**a**) the median RMS and MNF characteristics and (**b**) the RMS and MNF interquartile range (IQR).

Participant: SHR-F

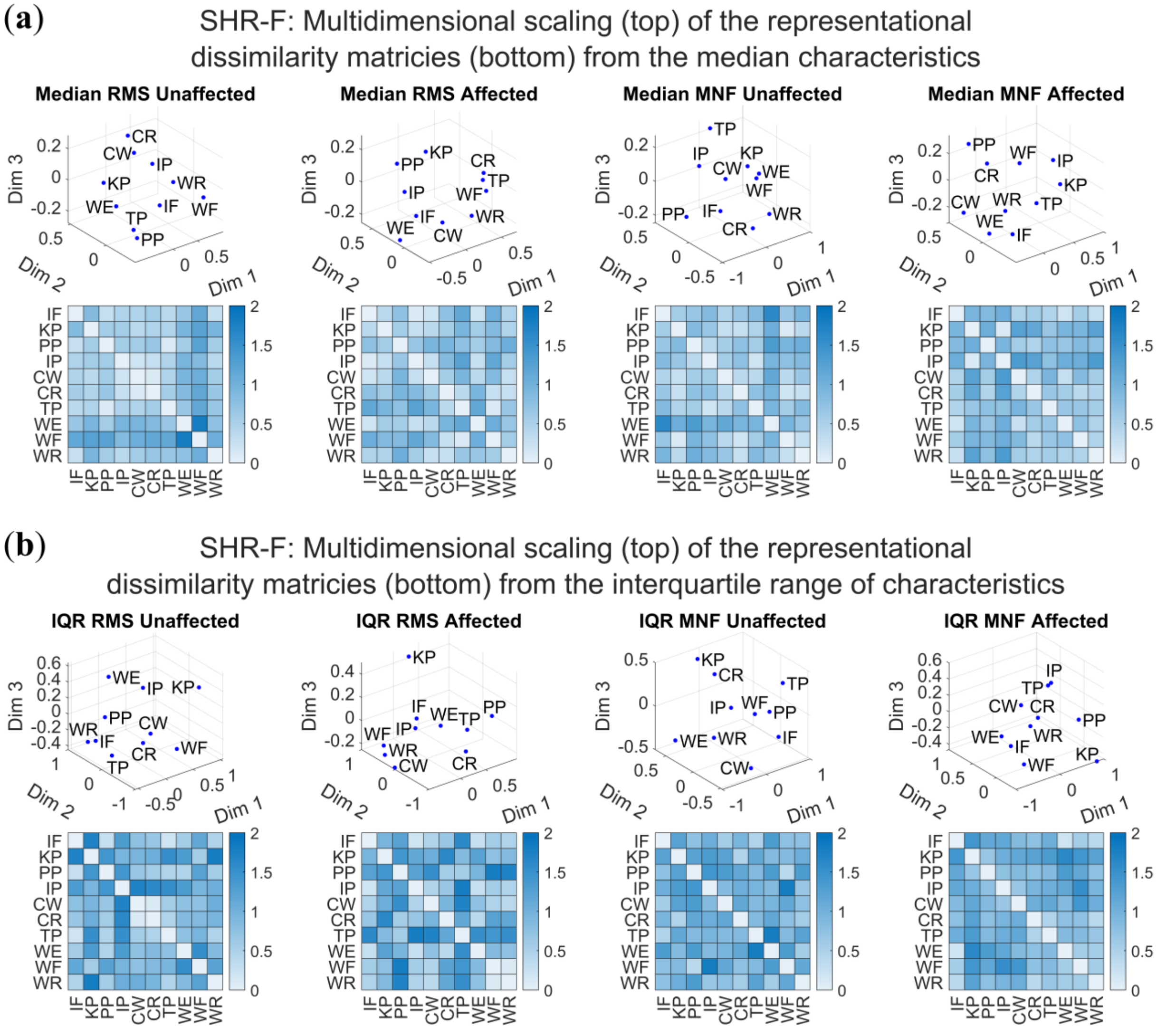

Supplementary Figure S24. Visual representation of the correlation distances between hand movements for the amplitude and spread of measurement characteristics of SHR-F. The multidimensional scaling plots are provided in three dimensions corresponding to the representational dissimilarity matrices for (**a**) the median RMS and MNF characteristics and (**b**) the RMS and MNF interquartile range (IQR).

Participant: SHR-G

(a)

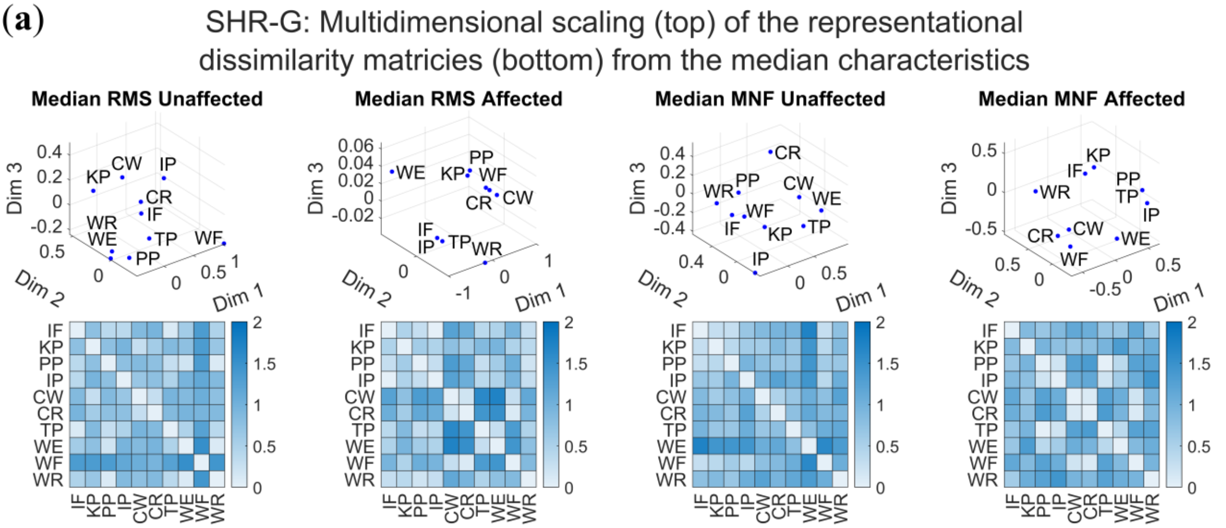

(b)

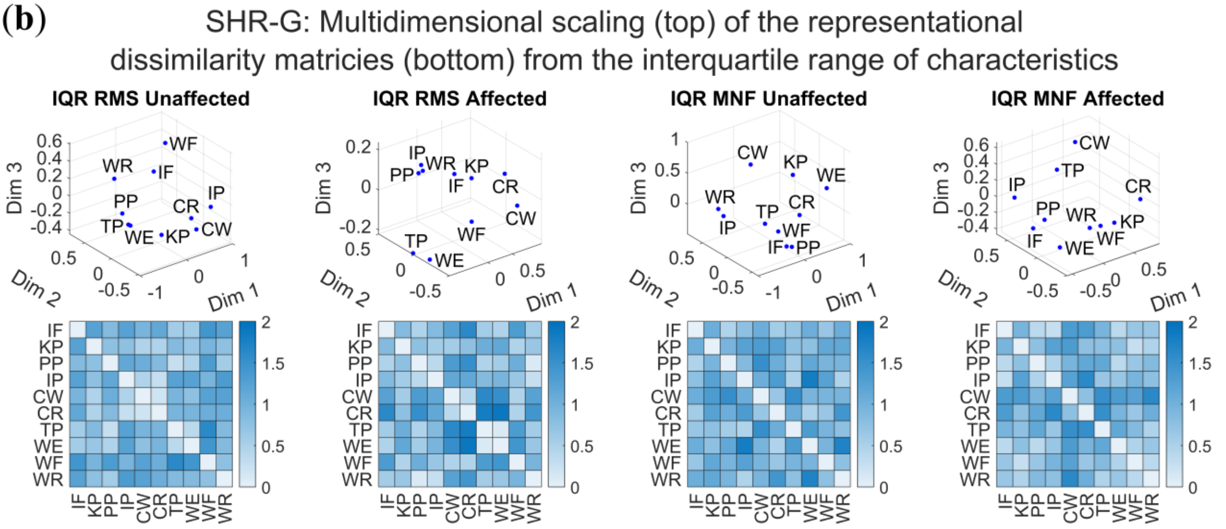

Supplementary Figure S25. Visual representation of the correlation distances between hand movements for the amplitude and spread of measurement characteristics of SHR-G. The multidimensional scaling plots are provided in three dimensions corresponding to the representational dissimilarity matrices for (**a**) the median RMS and MNF characteristics and (**b**) the RMS and MNF interquartile range (IQR).

Participant: SHR-H

(a)

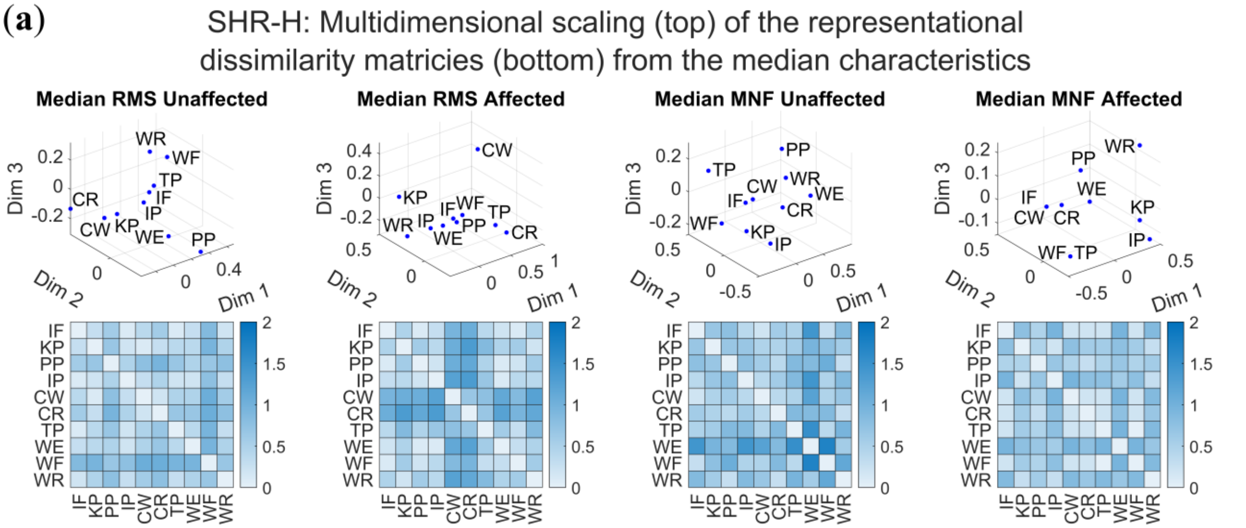

(b)

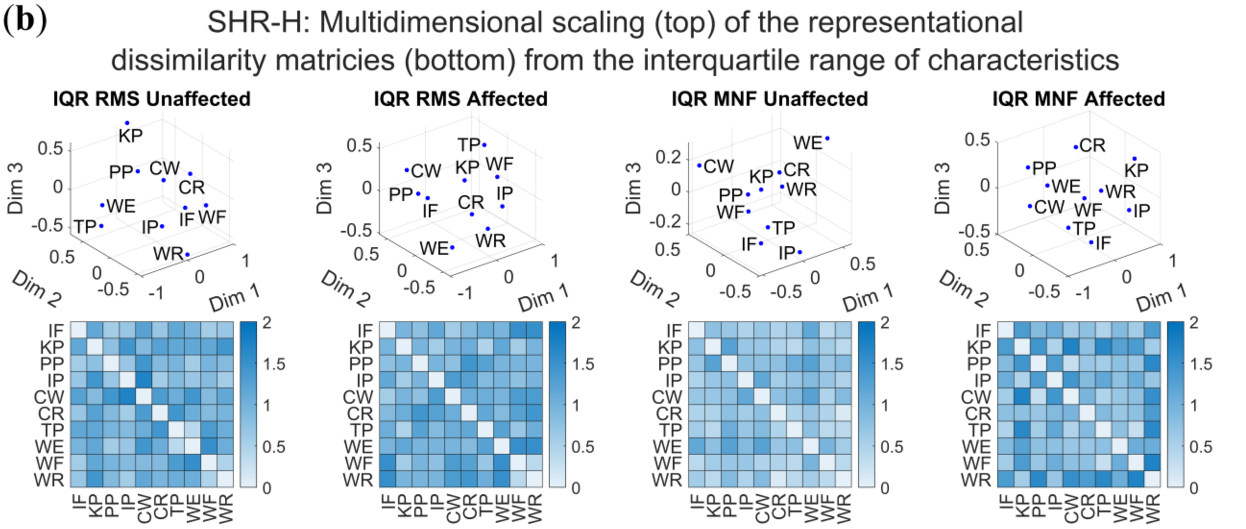

Supplementary Figure S26. Visual representation of the correlation distances between hand movements for the amplitude and spread of measurement characteristics of SHR-H. The multidimensional scaling plots are provided in three dimensions corresponding to the representational dissimilarity matrices for (**a**) the median RMS and MNF characteristics and (**b**) the RMS and MNF interquartile range (IQR).

Participant: SHR-I

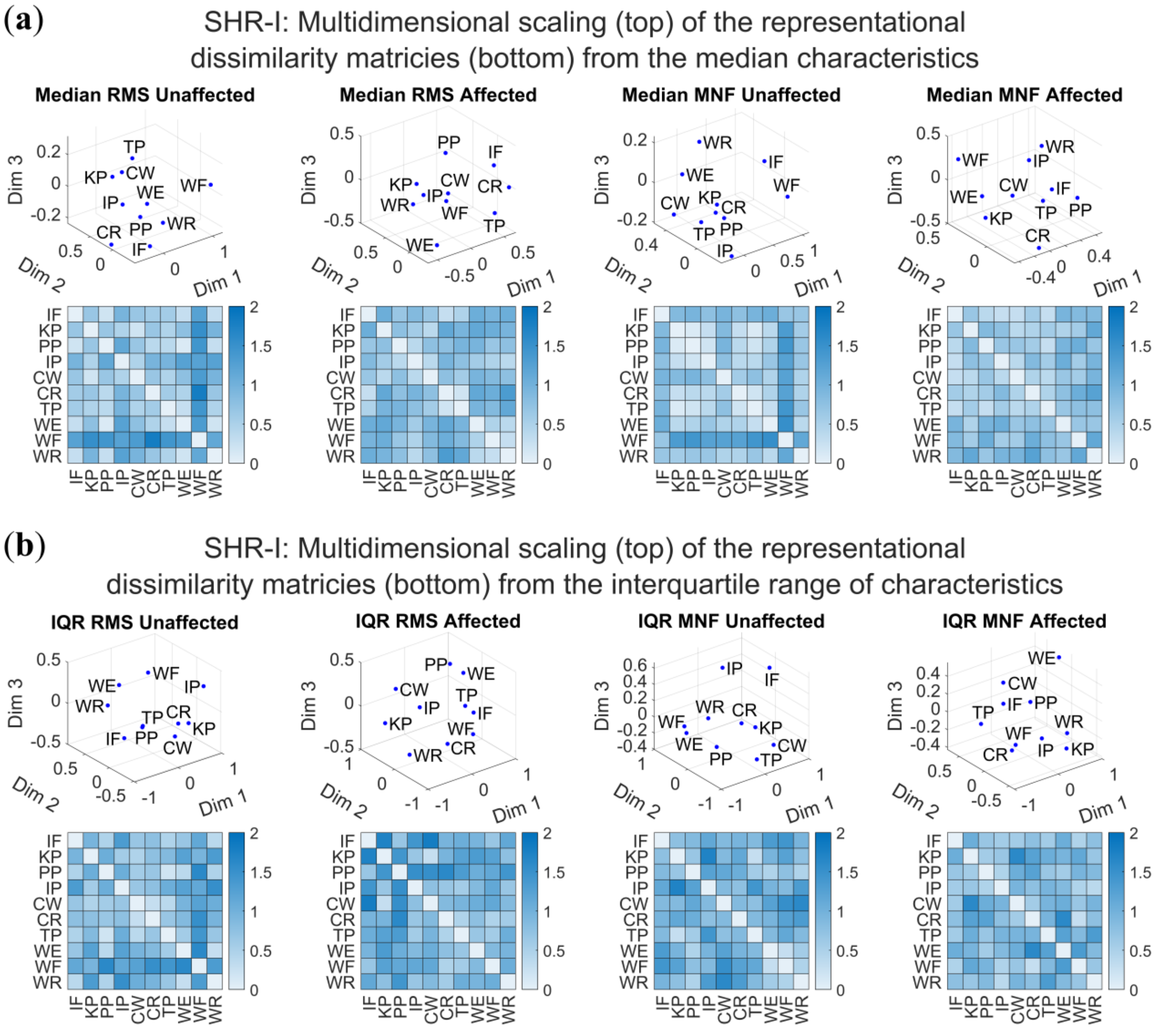

Supplementary Figure S27. Visual representation of the correlation distances between hand movements for the amplitude and spread of measurement characteristics of SHR-I. The multidimensional scaling plots are provided in three dimensions corresponding to the representational dissimilarity matrices for (**a**) the median RMS and MNF characteristics and (**b**) the RMS and MNF interquartile range (IQR).
